# FoxK1 and FoxK2 in insulin regulation of cellular and mitochondrial metabolism

**DOI:** 10.1038/s41467-019-09418-0

**Published:** 2019-04-05

**Authors:** Masaji Sakaguchi, Weikang Cai, Chih-Hao Wang, Carly T. Cederquist, Marcos Damasio, Erica P. Homan, Thiago Batista, Alfred K. Ramirez, Manoj K. Gupta, Martin Steger, Nicolai J. Wewer Albrechtsen, Shailendra Kumar Singh, Eiichi Araki, Matthias Mann, Sven Enerbäck, C. Ronald Kahn

**Affiliations:** 1 Sections of Integrative Physiology and Metabolism and Islet Cell Biology and Regenerative Medicine, Joslin Diabetes Center, Boston, MA 02215 USA; 2 Department of Medicine, Harvard Medical School, Boston, MA 02215 USA; 3 Department of Metabolic Medicine, Kumamoto University, 1-1-1 Honjo, Chuoku, Kumamoto 860-8556 Japan; 4 Department of Proteomics and Signal Transduction, Max Planck Institute of Biochemistry, 82152 Martinsried, Germany; 5 Department of Biomedical Sciences and NNF Centre for Basic Metabolic Research, Faculty of Health and Medical Sciences, University of Copenhagen, 2200 Copenhagen, Denmark; 6 Department of Clinical Proteomics, NNF Center for Protein Research, Faculty of Health and Medical Sciences, University of Copenhagen, 2200 Copenhagen, Denmark; 7 Department of Host Defense, The World Premier International Research Center Initiative Immunology Frontier Research Center, Osaka, 565-0871 Japan; 8 Department of Medical Biochemistry and Cell Biology, Institute of Biomedicine, University of Gothenburg, Medicinaregatan 9A, PO. Box. 440, 405 30 Göteborg, Sweden

## Abstract

A major target of insulin signaling is the FoxO family of Forkhead transcription factors, which translocate from the nucleus to the cytoplasm following insulin-stimulated phosphorylation. Here we show that the Forkhead transcription factors FoxK1 and FoxK2 are also downstream targets of insulin action, but that following insulin stimulation, they translocate from the cytoplasm to nucleus, reciprocal to the translocation of FoxO1. FoxK1/FoxK2 translocation to the nucleus is dependent on the Akt-mTOR pathway, while its localization to the cytoplasm in the basal state is dependent on GSK3. Knockdown of FoxK1 and FoxK2 in liver cells results in upregulation of genes related to apoptosis and down-regulation of genes involved in cell cycle and lipid metabolism. This is associated with decreased cell proliferation and altered mitochondrial fatty acid metabolism. Thus, FoxK1/K2 are reciprocally regulated to FoxO1 following insulin stimulation and play a critical role in the control of apoptosis, metabolism and mitochondrial function.

## Abstract (web summary)

Insulin signaling represses Forkhead transcription factor FoxO activity, which contributes to organismal metabolism. Here, the authors use proteomics to identify positively regulated insulin signaling targets FoxK1/K2 and demonstrate their role in lipid metabolism and mitochondrial regulation.

## Introduction

Insulin signals through the insulin receptor (IR) and to a lesser extent the insulin-like growth factor-1 receptor (IGF1R) to regulate a variety of cellular functions in multiple tissues, including gene transcription, glucose, lipid, and protein metabolism, as well as cell survival, growth control, and apoptosis^1–8^. The insulin and IGF1 receptor tyrosine kinases mediate their effects through tyrosine phosphorylation of substrate molecules, such as insulin receptor substrates-1 and substrates-2 (IRS-1 and IRS-2), leading to activation of two major pathways: the phosphoinositide 3-kinase (PI3K)-Akt pathway and the MAPK/ERK pathway^9^. The PI3K/Akt pathway activates several distinct downstream pathways and is central to most of the metabolic actions of insulin, whereas the MAPK pathway is more important in regulation of cell growth.

One action of Akt is to phosphorylate members of the FoxO family of Forkhead transcription factors (FoxO1, FoxO3, and FoxO4). This leads to the exclusion of FoxOs from the nucleus, thus blocking their transcriptional activity^10–14^. Extensive studies over the past decade have shown that turning off FoxOs, especially FoxO1 plays a significant role in insulin action and regulation of whole body energy metabolism. In the liver, the decrease in insulin action during fasting allows FoxO1 to enter the nucleus and promote the expression of the gluconeogenic enzymes G6pc (glucose-6-phosphatase, catalytic subunit) and PEPCK (phosphoenolpyruvate carboxykinase)^15–18^. FoxO1 also plays a key role in regulating adipocyte differentiation^19^ and in the insulin-mediated regulation of protein degradation in muscle^20^. Because insulin serves to negate the action of FoxOs by excluding these transcription factors from the nucleus, knockout of FoxO1 in liver or FoxO-1, FoxO-3, and FoxO-4 in muscle can reverse the effects of loss of insulin receptors and their effects on gene expression and metabolism in these tissues^20,21^.

Here, using a proteomics approach, we have identified two members of the FoxK family of Forkhead transcription factors, FoxK1 and FoxK2, as previously unrecognized targets of insulin action. By contrast to FoxO1, these transcription factors are translocated from the cytoplasm to the nucleus after insulin stimulation—a pattern that is reciprocal to that of FoxO1 after insulin stimulation. We show that activation of FoxK1 and FoxK2 after insulin stimulation is dependent on the mTOR and GSK3 pathways. Knockdown of FoxK1 and FoxK2 in a mouse hepatocyte cell-line causes marked alteration of the transcription of genes associated with lipid metabolism and mitochondrial functions. Thus, FoxK1/K2 represent critical components in IR and IGF1R-mediated signal transduction in controlling cell proliferation and metabolism.

## Results

### FoxK1 interacts with intracellular domains of IR and IGF1R

**Fig. 1:**
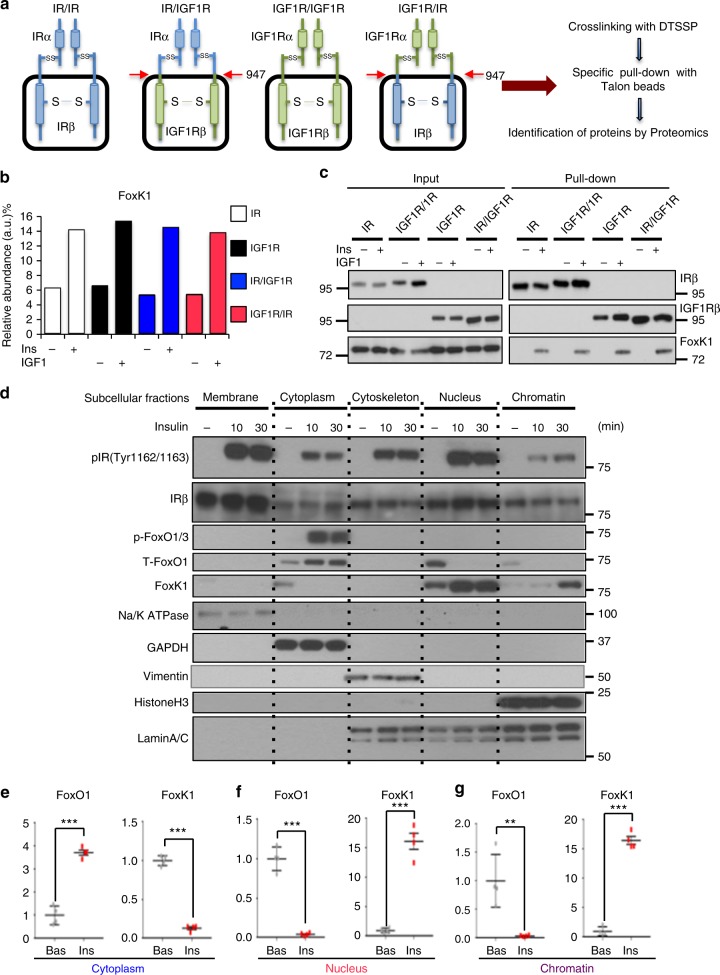
Identification of FoxK1 as a component of IR-mediated and IGFR-mediated signaling complex. **a** Schematic showing proteomic analysis using IR/IGF1R double-knockout preadipocytes reconstituted with normal IR, IGF1R, chimeric IR/IGF1R or IGF1R/IR before and after treatment of insulin/IGF-1. **b** Proteomics results indicating the relative abundance of FoxK peptides associated with immunoprecipitated receptors with or without 100 nM insulin/IGF-1 stimulation for 15 min. **c** His-tagged receptor-containing protein complexes were pulled down with Talon beads following insulin/IGF-1 stimulation and subjected to SDS-PAGE western blotting. Normal or chimeric receptors were detected using antibodies to the IRβ or IGF1Rβ subunits. Bound FoxK1 was detected using anti-FoxK1 antibody. **d** Subcellular fractions of cytoplasm, membrane, cytoskeletal, nucleus and chromatin were prepared from DKO brown preadipocytes re-expressing the IR before (0 min) and after 100 nM insulin for 10 and 30 min. FoxO1 and FoxK1 in each fraction was assessed by immunoblotting, as were markers for different fractions: membrane (IRβ), cytosol (GAPDH), nuclear and cytoskeletal (Lamin A/C) and chromatin (histone H3). **e**–**g** Quantitation of FoxO1 and FoxK1 in the cytoplasmic fractions (**e**), nuclear fractions (**f**) and chromatin fractions (**g**) 30 min after 100 nM insulin treatment as determined by scanning densitometry. (two-tailed Student *t*-test, *P < 0.05; **P < 0.01; ***P < 0.001, *n* = 4). All data are represented as mean ± SEM

To identify new components of IR and IGF1R signaling, we generated brown preadipocytes in which endogenous insulin and IGF-1 receptors had been genetically inactivated using Cre-lox recombination^2,22^. We then reconstituted the double knockout (DKO) cells with wild-type mouse 6XHis-tagged IR, IGF1R, or one of two chimeric receptors—one with the extracellular domain (ECD) of IR fused to the transmembrane and intracellular domains of the IGF1R (IR/IGF1R) or the ECD of IGF1R fused to the transmembrane and intracellular domains (ICD) of IR (IGF1R/IR) (Fig. 1a). To identify potential protein interactors, cells were stimulated with or without insulin or IGF-1 (depending on the extracellular domain) and treated with the crosslinking agent 3,3′-dithiobis(sulfosuccinimidyl propionate) (DTSSP, 1 mM). The 6XHis-tagged receptors and associated proteins were then pulled down with Talon beads (Fig. 1a). Mass spectroscopic proteomic analysis revealed a number of proteins that co-precipitated with each receptor construct, both in ligand stimulation-dependent and/or ligand stimulation-independent manners (Supplementary Fig. 1a). Among the proteins that associated with both receptors and chimeric receptors in a ligand stimulation-dependent manner was the Forkhead box protein FoxK1 (Fig. 1b). This association was confirmed by pulling-down the His-tagged receptors and immunoblotting for FoxK1 (Fig. 1c).

FoxK1 is a member of the K family of Forkhead transcription factors and is expressed in many organs and tissues in vertebrates species from fish to human (Supplementary Fig. 1b,c). Previous studies have shown that FoxK1 can shuttle between the cytoplasm and the nucleus in skeletal muscle following cells starvation^23^. To assess effects of insulin on subcellular localization of FoxK1, DKO preadipocytes expressing human insulin receptors were stimulated with insulin then lysed and fractionated by differential centrifugation into membrane (marked by Na/K ATPase), cytoplasm (marked by GAPDH), cytoskeleton (marked by vimentin), nuclear (marked by lamin A/C), and chromatin (both lamin A/C and histone H3 positive) fractions. The levels of FoxK1 and FoxO1 in each fraction were determined by immunoblotting (Fig. 1d and Supplementary Fig. 1d, e). As previously described^13^, FoxO1 was predominantly localized in the nucleus at the basal state (0 min) and, following insulin stimulation, was phosphorylated and translocated into the cytoplasm (Fig. 1d and Supplementary Fig. 2a). In contrast, while FoxK1 protein could be detected in both the cytoplasm and nucleus in unstimulated cells, by 30 min following insulin stimulation, cytoplasmic FoxK1 shuttled from the cytoplasm to the nucleus and chromatin fractions. Interestingly, phosphorylated IR-β subunit was also detected in the nucleus and chromatin fractions, consistent with some previous reports that IR can translocate to the nucleus following insulin stimulation^24,25^. These changes in FoxO1 and FoxK1 in each fraction are quantitated in Fig. 1e–g. This reciprocal pattern of translocation of FoxO1 and FoxK1 was also observed in wild-type brown preadipocytes expressing only endogenous IR (Supplementary Fig. 2b). Thus, opposite to FoxO1, following insulin stimulation FoxK1 is translocated from the cytoplasm to the nucleus and interacts with chromatin.

### Insulin-induced FoxK1 translocation is dependent on Akt

**Fig. 2:**
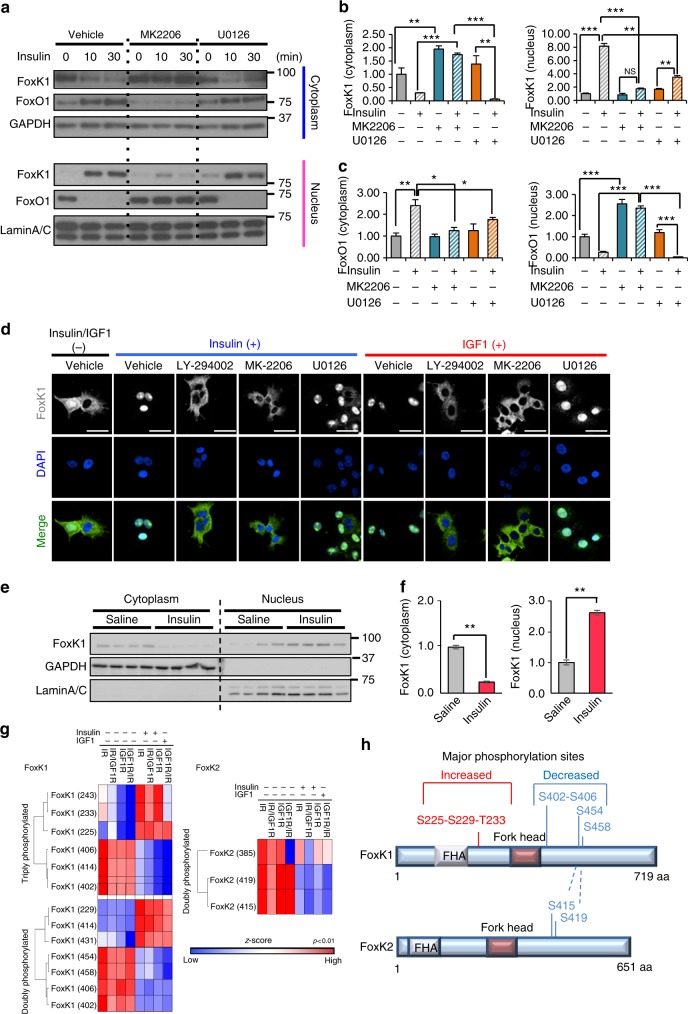
Insulin and IGF-1 regulate nuclear translocation of FoxK1 in an Akt-dependent manner. **a** Immunoblotting of FoxO1 and FoxK1 in nuclear and cytoplasmic fractions extracted from IR-expressing brown preadipocytes before and after stimulation with 10 nM insulin at the indicated times in the presence or absence of the Akt inhibitor MK2206 (5 μM) or the MEK1/2 inhibitor U0126 (20 μM). GAPDH is a cytosolic marker, and Lamin A/C is a nuclear marker. **b**, **c** Densitometry of FoxK1 (**b**) and FoxO1 (**c**) in the cytoplasmic fractions and nuclear fractions 30 min after insulin stimulation as in Supplementary Fig. 3a. (One-way ANOVA followed by Tukey-Kramer post hoc analysis, *P < 0.05; **P < 0.01; ***P < 0.001, *n* = 3). All data are represented as mean ± SEM. **d** Representative images of AML 12 cells immunostained for FoxK1 and DAPI before and 30 min after 100 nM insulin treatment in the presence or absence of 50 μM PI3K inhibitor LY-294002 or 5 μM MK2206 (Akt inhibitor) or 20 μM U0126 (MEK inhibitor). DAPI was used to label the nucleus. Scale bars, 50 μm. **e** Immunoblotting of FoxK1 in nuclear and cytoplasmic fractions extracted from 2-month-old C57BL/6 J mice liver tissues 15 min after injection of saline or 5 U insulin via the inferior vena cava. GAPDH is a cytosolic marker, and Lamin A/C is a nuclear marker. **f** Densitometry of FoxK1and FoxO1 in the cytoplasmic fractions (left) and nuclear fractions (right) 15 min after insulin stimulation as in Fig. 2e (two-tailed Student *t*-test, *P < 0.05; **P < 0.01; ***P < 0.001, *n* = 4). All data are represented as mean ± SEM. **g** Heatmap of Log2 transformed, *z*-score phosphosite intensities for FoxK1 and FoxK2 in the presence or absence of insulin/IGF-1 in cells expressing normal or chimeric receptors. Significant increase or decrease of the phosphorylation clusters for FoxK1 and FoxK2 after insulin/IGF-1 stimulation are shown in panel **h**

To define the upstream signaling pathways involved in regulation of FoxK1, we analyzed FoxK1 translocation in brown preadipocytes upon insulin stimulation in the presence of the Akt inhibitor MK2206, the PI 3-kinase inhibitor (LY294002) or the MAPK/ERK (MEK1/2) inhibitor U0126. Using cell fraction, in the presence of the Akt inhibitor, FoxK1 was retained in the cytoplasm and inhibited from translocation into the nucleus at 10 and 30 min, whereas the MEK1/2 inhibitor produced only minimal effects on cytoplasmic versus nuclear localization of FoxK1 in the basal state or after insulin stimulation (Fig. 2a, b). Likewise, FoxO1 shuttling from the nucleus to the cytoplasm following insulin stimulation was blocked by inhibition of PI3K and Akt, but not significantly altered by MEK/ERK inhibition (Fig. 2a, c and Supplementary Fig. 3a, b). Translocation of FoxK1 to the nucleus was also observed in response to EGF and to a lesser extent PDGF, and like the effect of insulin and IGF-1, this translocation was blocked by inhibition of PI3K (Supplementary Fig. 3b). Two-photon immunofluorescence microscopic analysis of AML12 liver cells confirmed these findings. Thus, in the basal state, most cells exhibited a predominantly cytoplasmic localization of FoxK1 (Fig. 2d, vehicle), whereas following insulin or IGF-1 stimulation almost all FoxK1 in AML12 cells was in the nucleus, and this nuclear localization was inhibited by either of two PI3K/Akt inhibitors (LY-294002 and MK2206), but not by the MAPK pathway inhibitor U0126 (Fig. 2d and Supplementary Fig. 3c, d, e). FoxK1 translocation from the cytoplasm to the nucleus was also observed in liver tissue following in vivo insulin stimulation (Fig. 2e, f).

To identify the insulin-dependent phosphorylation sites on FoxK1, we performed a global phosphoproteomics analysis^26^ of cells expressing either the native or the chimeric versions of IR and IGF1R with or without insulin/IGF1 stimulation for 15 min. For this analysis, phosphopeptides were enriched prior to MS using TiO_2_. We identified two clusters of phosphorylation sites in FoxK1 and/or FoxK2 that were significantly modulated in a ligand-dependent and receptor-dependent manner (Fig. 2g, h). One was a more N-terminal cluster of sites including Ser225/S229/T233 in FoxK1 that increased following ligand stimulation. The other was a more C-terminal cluster of phosphorylation sites found in both FoxK1 (S402/S406 and S454/S458) and FoxK2 (S415/S419). This cluster showed decreased upon insulin stimulation (Fig. 2g, h and Supplementary Fig. 3e) or in some cases a changing pattern of phosphorylation. For example, in this cluster, the intensity of the triply phosphorylated peptide containing S414 of FoxK1 was decreased after ligand stimulation (Fig. 2g; left upper), while the level of the doubly phosphorylated S414-FoxK1 peptide was increased by ligand stimulation (Fig. 2g; left lower).

### FoxK1/K2 nuclear translocation is also regulated by GSK3α/β

**Fig. 3:**
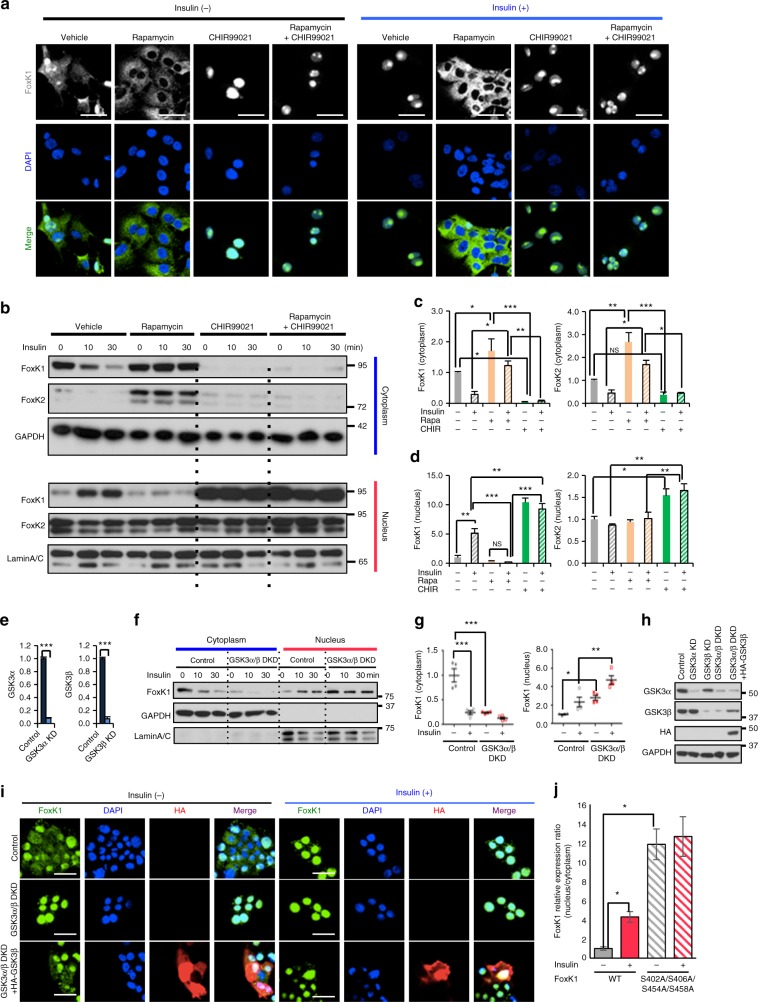
FoxK1/K2 nuclear translocation is induced by GSK3α/β inactivation. **a** Immunostaining of FoxK1 before and 30 min after 100 nM insulin in the presence or absence of rapamycin (100 nM) or/and CHIR99201 (10 μM) in AML12 cells. Scale bars, 50 μm. **b** Nuclear and cytoplasmic fractions extracted from the AML12 cells before and after 100 nM insulin as indicated time points in the presence or absence of 100 nM rapamycin or/and 10 μM CHIR99201. FoxK1/K2 in each fraction was assessed by immunoblotting. **c**, **d** Quantification of FoxK1/K2 intensity in the indicated fractions (**c**, **d**) before and 30 min after insulin in the presence or absence of rapamycin or/andCHIR99201 as shown in Supplementary Fig. 4a. (One-way ANOVA followed by Tukey-Kramer post hoc analysis, *P < 0.05; **P < 0.01; ***P < 0.001, *n* = 3). **e** Quantification of immunoblot (Supplementary Fig. 4b, c) in AML12 cells transfected with Control siRNA or siRNAs for GSK3α or GSK3β cells. (two-tailed Student *t*-test, ***P < 0.001). (*n* = 4). Data are represented as mean ± SEM. **f** Nuclear and cytoplasmic fractionation and immunoblotting of FoxK1 extracted from the indicated cells before and after 100 nM insulin as 10 min and 30 min. **g** Quantitation of FoxK1 in theindicated fractions in Supplementary Fig. 4a (One-way ANOVA followed by Tukey-Kramer post hoc analysis, *P < 0.05; **P < 0.01; ***P < 0.001, *n* = 3). Data are represented as mean ± SEM. **h** Immunoblotting with indicated antibody in AML12 cells transfected with NS siRNA, GSK3α siRNA, GSK3β siRNA, GSK3α/β DKD, and re-expressed with HA-tagged GSK3β in GSK3α/β DKD cells. **i** Immunostaining of FoxK1 before and 30 min after 100 nM insulin in Control, GSK3α/β double knockdown cells and re-expressed with HA-GSK3β in GSK3α/β DKD cells. Scale bars, 50 μm. **j** Nuclear and cytoplasmic fractionation were extracted from cells overexpressing 3XFlag-FoxK1 wild type or the 3XFlag-FoxK1 S402A/S406A/S454A/S458A mutant before and after 100 nM insulin for 30 min. The relative FoxK1 nucleus/cytoplasm protein expression ratio was quantified in Supplementary Fig. 4h. (two-tailed Student *t*-test, *P *<* 0.05, *n* = 3)

The phosphorylation sites in all clusters in FoxK1 (S225/S229/T233, S402/S406, S454/S458) and FoxK2 (S415/S419) showed the motif S-x-x-x-S (Supplementary Fig. 3d), corresponding to the consensus S/T-x-x-x-S/T motif of GSK3^27^. Previous reports, on the other hand, have suggested that the translocation of FoxK1 from the nucleus to the cytoplasm that occurs with serum starvation in C2C12 muscle cells is dependent on mTOR signaling^25^. To more directly determine if mTOR and/or GSK3 were involved in the regulation of FoxK1 translocation by insulin, AML12 cells were serum-starved for 3 h and stimulated with 100 nM insulin or vehicle for 30 min in the absence or presence of the GSK3α/β inhibitor CHIR99201 or the mTOR inhibitor rapamycin (Fig. 3a). Rapamycin completely inhibited FoxK1 nuclear localization both before and after insulin treatment. Cells treated with the GSK3 inhibitor CHIR99021, on the other hand, showed strong FoxK1 nuclear localization even in the absence of insulin treatment, indicating that even basal GSK3 activity is critical for the retention of FoxK1 in the cytoplasm. Addition of CHIR99021 and rapamycin simultaneously produced effects similar to CHIR99021 alone (Fig. 3a). These results were confirmed by Western blot analysis (Fig. 3b and Supplementary Fig. 4a). By comparison to FoxK1, FoxK2 appears to localize to a greater extent in the nucleus rather than the cytoplasm even before insulin stimulation (Fig. 3b and Supplementary Fig. 4a). Thus, both FoxK1 and FoxK2 were retained in the cytoplasm in the presence of rapamycin, with the translocation into the nucleus being more affected for FoxK1 than FoxK2. Treatment with the GSK3 inhibitor CHIR99021, on the other hand, caused a marked accumulation of both FoxK1 and FoxK2 in the nucleus irrespective of insulin stimulation. These results are quantitated in Fig. 3c, d.

To further confirm the role of GSK3 in FoxK localization, we knocked-down GSK3α and GSK3β using RNAi. This produced ~90% reduction of GSK3α/β at the protein level (Fig. 3e and Supplementary Fig. 4b, c). Knockdown of GSK3α and GSK3β markedly reduced the cytoplasmic localization of FoxK1 and increased its nuclear localization (Fig. 3f, g and Supplementary Fig. 4d). This effect of GSK3α/β knockdown on nuclear localization of FoxK1 can be rescued by overexpression of HA-tagged GSK3β cDNA (Fig. 3h, i and Supplementary Fig. 4e). Taken together these inhibitor and knockdown studies demonstrate that translocation of FoxK1 to the nucleus is dependent on the Akt-mTOR pathway, while its localization to the cytoplasm in the basal state is dependent on the serine/threonine protein kinase GSK3. When lysates from insulin stimulated AML12 cells and liver samples were immunoprecipitated with anti-p-Tyr antibody, we could not find any evidence of Tyr phosphorylation of FoxK1 by western blotting (Supplementary Fig. 4f, g). On the other hand, serine-to-alanine substitutions in the GSK3 phosphorylation motifs (S402A/S406A, S454A/S458A) in FoxK1 resulted in markedly reduced cytoplasmic and increased its nuclear localization of FoxK1 as compared with ectopically expressed wild type (WT) FoxK1 (Fig. 3j and Supplementary Fig. 4h).

### FoxK1/K2 as a modifier of insulin signal transduction

**Fig. 4:**
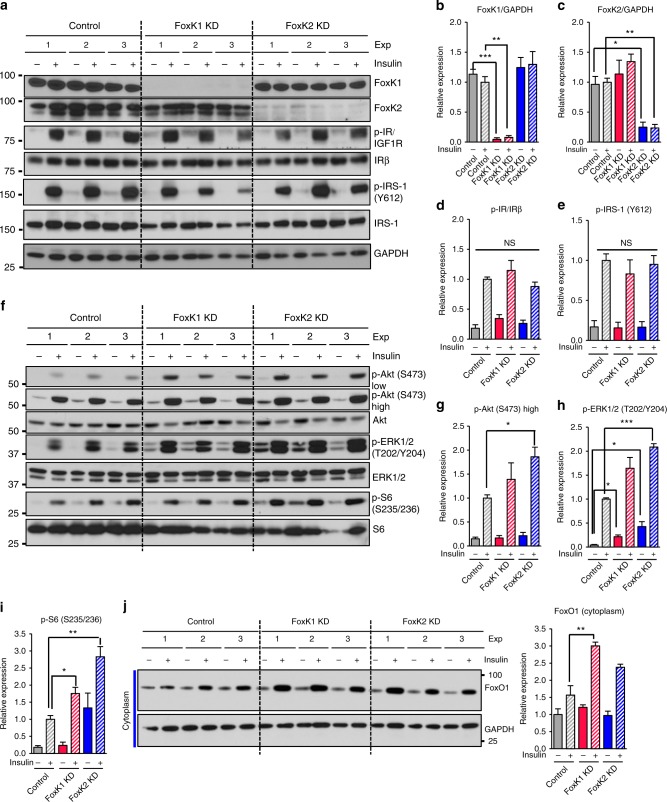
Role of FoxKs in the regulation of insulin-mediated signal transduction. **a** Immunoblotting for phosphorylation of IR and IRS-1 in lysates from Control (NS siRNA) AML12 cells or cells depleted of either FoxK1 (FoxK1 KD) or FoxK2 (FoxK2 KD) cells by siRNAs stimulated with 100 nM insulin for 5 min. **b**–**e** Densitometric analysis of FoxK1, FoxK2, phosphorylated IR and IRS-1 following 5 min stimulation. Data are mean ± SEM (One-way ANOVA followed by *t*-test with Bonferroni correction, *P < 0.05; **P < 0.01; ***P < 0.001, *n* = 3). **f** Immunoblotting of the phosphorylation of Akt, ERK, and S6 in lysates from Control (NS siRNA) AML12 cells or cells depleted of either FoxK1 (FoxK1 KD) or FoxK2 (FoxK2 KD) cells by siRNAs and stimulated with 100 nM insulin for 5 min. **g**–**i** Densitometric analysis of phosphorylated Akt, ERK, and S6 following 5 min insulin stimulation. Data are mean ± SEM (One-way ANOVA followed by *t*-test with Bonferroni correction, *P < 0.05; **P < 0.01; ***P < 0.001, *n* = 3). **j** Cytoplasmic fractionation and immunoblotting of FoxK1 and FoxK2 extracted from the AML12 cells before and after 100 nM insulin as indicated time points (0 min, 15 min)) in the Control, FoxK1 KD and FoxK2 KD cells (left) and quantitation by scanning densitometry (right). Data are mean ± SEM (One-way ANOVA followed by *t*-test with Bonferroni correction, *P < 0.05; **P < 0.01; ***P < 0.001, *n* = 3)

To better define the role of FoxK1 and FoxK2, we performed knockdown of both molecules in AML12 cells using RNAi. As expected, knockdown of either FoxK1 (FoxK1 KD) or FoxK2 (FoxK2 KD) did not affect expression or tyrosine phosphorylation of IR or IRS1 (Y612) (Fig. 4a–e). Somewhat surprisingly, however, in both FoxK1 KD and FoxK2 KD cells, insulin-stimulated phosphorylation of Akt (S473), ERK1/2 (T202/Y204), and ribosomal S6 protein (S235/S236) were all increased by 1.5-fold to 3-fold (Fig. 4f–i). The enhanced activation of Akt by FoxK1 or FoxK2 knockdown was associated with enhanced the shuttling of FoxO1 to the cytoplasm in response to insulin (Fig. 4j). Conversely, overexpression of FoxK1 and FoxK2 resulted in reduced phosphorylation of ERK1/2 (T202/Y204) and ribosomal S6 protein (S235/S236), but did not affect expression or phosphorylation of IR/IGF1R, IRS-1 (Y612), or Akt (S473) (Supplementary Fig. 5a–i). Thus, not only is the nuclear translocation of FoxK1/K2 regulated by insulin, but FoxK1/K2 also play a regulatory role in insulin signal transduction and can modify signaling through Akt, ERK, S6K, and FoxO1.

### Regulation of gene expression by loss of FoxK1 and FoxK2

**Fig. 5:**
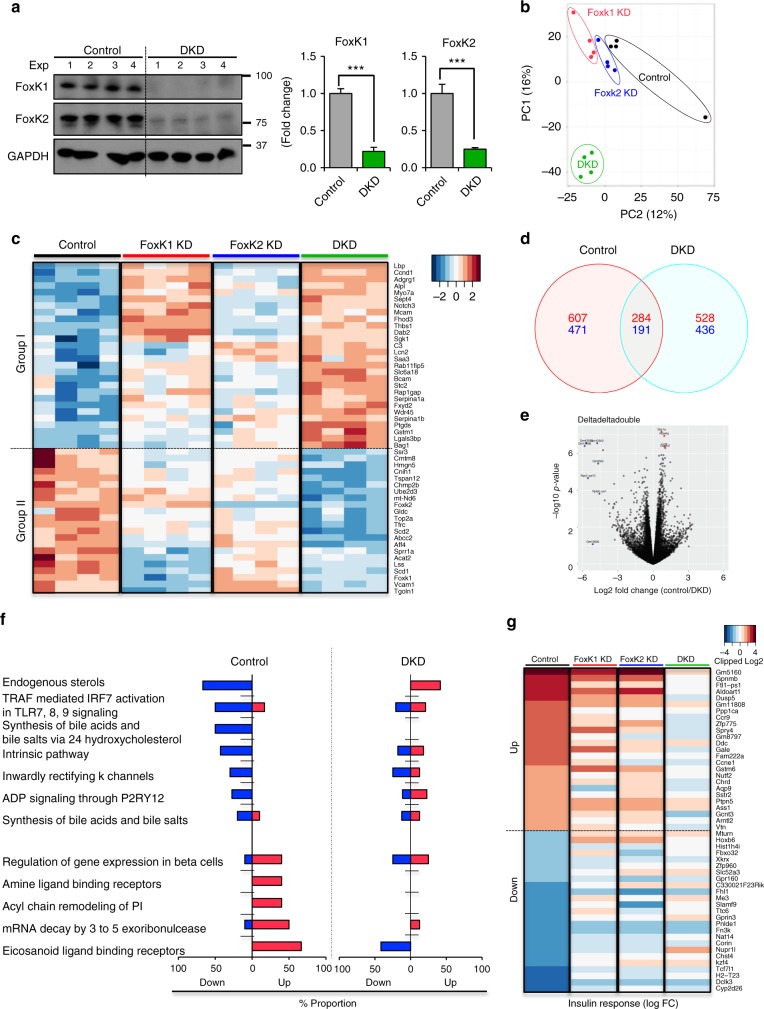
Role of FoxKs in the regulation of gene expression. **a** Immunoblotting of FoxK1 and FoxK2 from lysates of Control (NS siRNA) and FoxK1/K2 double knockdown (DKD) cells using siRNAs (two-tailed Student *t*-test, *P < 0.05; **P < 0.01; ***P < 0.001) (*n* = 4). All data are represented as mean ± SEM. **b** PCA plots of the transcriptome profiles of Control (Black), FoxK1 KD (Red), FoxK2 KD (Blue), and DKD (Green) cells. **c** Heatmap of top 50 most significantly changed genes among all groups. **d** Venn diagram showing the numbers of significantly regulated genes by insulin in Control and DKD cells (FDR < 0.25). **e** Volcano plot showing the distribution of differentially regulated genes by insulin stimulation with log-fold change in Control versus DKD fold change on *X*-axis and −log10 *P* value on *Y*-axis. **f** Top directionally regulated reactome pathways by insulin in Control but not DKD cells. **g** Heatmap of top 50 (up and down: each top 25 for each) differentially regulated by insulin in Control and DKD cells

To define the roles of FoxK1 and FoxK2 in gene expression, FoxK1 KD, FoxK2 KD, and FoxK1/FoxK2 double knockdown (DKD) AML12 cells (confirmed by western blot analysis Fig. 5a and Supplementary Fig. 6a) were serum-starved, and then stimulated with insulin (100 nM) or vehicle for 6 h. Total mRNAs from these cells were subjected to RNAseq analysis using an Illumina HiSeq 2500 platform. Principle component analysis demonstrated the global change in gene expression of with both individual knockdowns and especially the DKD cells (Fig. 5b). In the basal state, of over 13,000 transcripts detected, 5974 genes were differentially expressed in DKD cells, with 2996 upregulated and 2978 downregulated, compared with the Control (FDR < 0.25, Supplementary Fig. 6b). Among these, 358 upregulated genes and 378 downregulated genes were selective to FoxK1 KD, and 84 genes were selectively upregulated and 57 were downregulated in Foxk2 KD cells (Supplementary Fig. 6b). Gene set pathway analysis indicated that genes related to cell proliferation, cell metabolism, and mitochondrial metabolism were downregulated in DKD cells compared with Control, while genes of the apoptosis pathway were upregulated (Supplementary Fig. 6c and Supplementary Table 1). A heatmap of the top 50 differentially expressed genes in DKD cells fell into two distinct clusters (Fig. 5c and Supplementary Table 2). Twenty-eight genes (*Group I*) were upregulated in DKD cells, including cyclin D1 (*Ccnd1*), serum/glucocorticoid regulated kinase 1 (*Sgk1*), adhesion G protein-coupled receptor G1 (*Adgrg1*), melanoma cell adhesion molecule (*Mcam1*), alkaline phosphatase, liver/bone/kidney (*Alpl*), and the WD-repeat domain phosphinositide-interacting protein 4 (*Wdr45*), a molecule associated with autophagy. Twenty-two genes were highly downregulated in DKD cells (*Group II*). These were mostly genes associated with lipid metabolism, including stearoyl-CoA desaturase-1 and desaturase-2 (*Scd1 and Scd2*), mitochondrially encoded NADH dehydrogenase 6 (*mt-ND6*), vascular cell adhesion molecule (*Vcam1*), AF4/FMR2 family member 4 (*Aff4*), trans-golgi network protein (*Tgoln1)*, and of course, *Foxk1* and *Foxk2*. To determine the effect of FoxK1 in mice, we also induced deletion of FoxK1 in liver by tail vein injections of adenovirus encoding GFP (Control) or Cre into FoxK1^flox/flox^ mice. Interestingly deletion of FoxK1 in liver caused the decrease in expression of *Vcam1*, *Aff4, and Tgolin1* mRNAs and conversely increased in expression of *Mcam, Adgrg1, and Sgk1* mRNAs liver (Supplementary Fig. 6d).

Knockdown of FoxK1 and FoxK2 also affected gene expression after 6 h insulin stimulation. In Control AML12 cells, 891 transcripts were upregulated (indicated in red numbers) and 662 transcripts were downregulated (indicated in blue numbers) by insulin (at an FDR < 0.25, |FC| > 1.5) (Fig. 5d). These included a number of metabolic genes, other protein-coding genes and non-coding genes, i.e., pseudogenes, miRNAs, and snoRNAs. DKD cells showed a similar number of changes after insulin stimulation, with only approximately 30% of insulin upregulated and insulin downregulated genes being similarly regulated in both Control and DKD cells (Fig. 5d, e). Gene set analysis showed that insulin stimulation induced upregulation of genes involved in classical metabolic pathways, such as glycolysis, glucose metabolism, cholesterol biosynthesis, and genes regulated in diabetes in both Control and DKD AML12 cells (Supplementary Table 3). Interestingly, DKD cells showed the blunted regulation of other pathways upon insulin stimulation, including eicosanoid ligand binding receptors, mRNA decay acyl chain remodeling and amine ligand binding receptors, endogenous sterols and synthesis of bile acids and bile salts via 24-hydroxycholesterol (Fig. 5f and Supplementary Fig. 6e). A heat map and list of the top 50 differentially regulated genes by insulin at the 6 h time point among all groups, including a comparison between Control vs. DKD, is shown in Fig. 5g, Supplementary Fig. 6f and Supplementary Table 4. This revealed significant changes in several genes associated with glucose and lipid metabolism: 6-phosphofructose 2 kinase (*1810024B03Rik*), radical S-adenosyl methionine domain containing 2 (*Rsad2*), dual specificity phosphatase 5 (*Dusp5*), apolipoprotein C4 (*Apoc4*), glutathione S-transferase mu6 (*Gstm6*), arginosuccinate synthase 1 (*Ass1*), aldolase 1A (*Aldoart1*), DOPA carboxylase (*Ddc*), ubiquitin-specific peptidase 17 line A (*Usp17la*), methionine adenosyltransferase 1A (*Mat1a*), glucosaminyl (N-acetyl) transferase 3, mucin type (*Gcnt3*), aquaporin 9 (*Aqp9*), UDP glucose 4 epsilon (*Gale*), hexokinase 2 (*Hk2*), and the cystine/glutamate transporter (*Slc7a11*).

### FoxK1/2 regulate mitochondrial β-oxidation and biogenesis

**Fig. 6:**
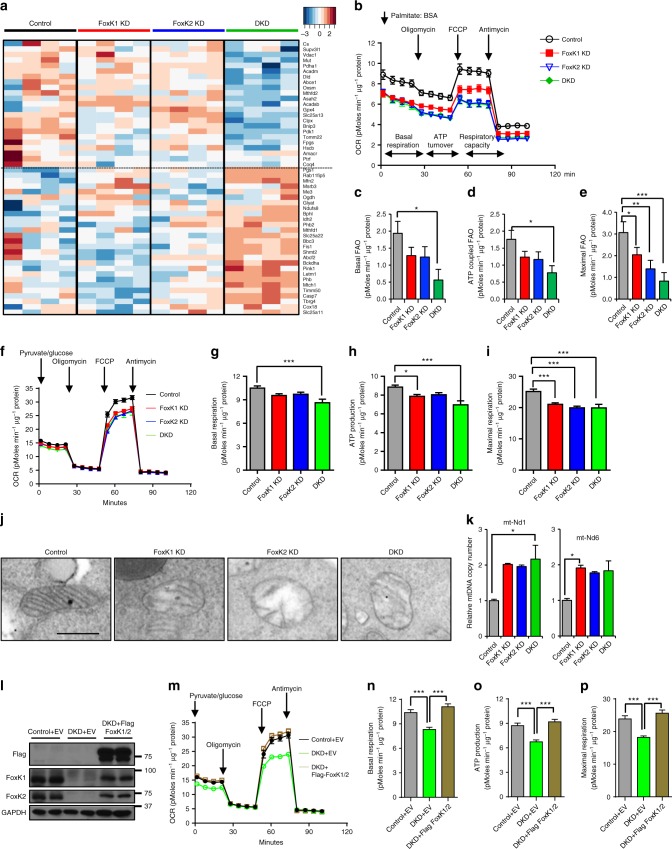
Role of FoxKs in the regulation of FAO and mitochondrial biogenesis. **a** Heatmap of mitochondrial-related gene expression in all groups. **b** Measurement of FAO) using Seahorse Bioanalyzer. **c**–**e** Quantitation of basal (**c**), ATP-coupled (**d**) and maximal (**e**) OCRs of FAO. Error bars represent SEM. (One-way ANOVA followed by post hoc analysis, *P < 0.05; **P < 0.01; ***P < 0.001, Control, *n* = 7, FoxK1 KD, *n* = 8, FoxK2 KD, *n* = 9 and DKD, *n* = 8). **f** Mitochondrial oxidative phosphorylation activity in Control, Foxk1 KD, Foxk2 KD, and DKD cells. Quantitation of **g** basal respiration, (**h**) ATP production, and (**i**) maximal respiration capacity as measured by OCR. Error bars represent SEM. (One-way ANOVA followed by Dunnett’s post hoc analysis, *P < 0.05; **P < 0.01; ***P < 0.001, Control, *n* = 14, FoxK1 KD, *n* = 14, FoxK2 KD, *n* = 13 and DKD, *n* = 13). **j** Representative electron microscopic images of mitochondria in Control, FoxK1 KD, FoxK2 KD and DKD AML 12 cells. Scale bar, 0.5 μm. **k** Mitochondrial DNA copy number was assessed by qPCR of mt-ND1/mt-ND6 and normalized to genomic DNA encoding GAPDH in extracted total DNA. (One-way ANOVA followed by post hoc analysis, *P < 0.05; **P < 0.01; ***P < 0.001, *n* = 4). **l** Immunoblotting with anti-FoxK1, FoxK2, and Flag antibody in lysates from Control (NS siRNA), DKD and DKD cells re-expressing Flag-tagged FoxK1/K2. **m** Mitochondrial oxidative phosphorylation activity in Control, DKD cells and DKD cells re-expressing Flag-FoxK1/K2 measured by Seahorse Bioanalyzer. 25 mM glucose and pyruvate mix was used as substrate. Quantification of basal respiration (**n**), ATP production (**o**) and maximal respiratory capacity (**p**) as measured by OCR. Error bars represent SEM. (One-way ANOVA followed by Dunnett’s post hoc analysis, *P < 0.05; **P < 0.01; ***P < 0.001, Control + EV, *n* = 13, DKD + EV, *n* = 13 and DKD + Flag-FoxK1/K2, *n* = 12)

Transcriptome analysis in the DKD cells revealed clear alterations in genes involved in mitochondrial metabolism including *Tomm22, Mfn2, Letm1*, and *Mtch1* (Fig. 6a, Supplementary Fig. 6c and Supplementary Tables 5, 6). In addition, DKD cells showed increased expression of multiple genes associated with mitochondrial oxidative phosphorylation, such as components of NADH dephosphorylase 1 subunit complex (*Ndufs8, Ndufb3, Ndufa11, Ndufv1, Ndufv9, Ndufc2*, and *Ndufb7*); ATPase H^+^ transforming subunits (*Atp6v1g1, Atp6v1b2, Atp6v1f, Atp6v0b, Atp6v0a1, Atp6v0a2*, and *Atp6v0a4*); ATP synthases (*ATP5o*, and *ATP5g3*); cytochrome C oxidase subunits (*Cox6b1, Cox7a1, Cox10*, and *Cox6b1*); succinate dehydrogenase complexes (*Sdha, Sdhd, Sdhb, Sdhc*); and ubiquinol cytochrome C reductase binding proteins (*Uqcrq*). These genes which are upregulated in the DKD cells are presumably normally downregulated by the action of FoxK1 and FoxK2. Interestingly, DKO cells showed marked downregulation of other mitochondrial regulatory genes, including other subunits of the NADH dephosphorylase 1 subunit complex (*Ndub2, Ndufs2, Ndufs1, Ndufv2, Ndufa5*, and *Ndufa1*); cytochrome C1 and cytochrome oxidase subunits (*Cox6a1, Cox7c, Cox5a, Cox17* and *Cox7b*); ATPase H^+^ transforming subunits (Atp5g1, Atp6v1e1, Atp6v1d, and Atp6v0d1); ATP synthases (*ATP6v0c, ATP5g1* and *ATP5e*); pyrophosphatase (*Ppa2*) and some components of the ubiquinol cytochrome C reductase complex (*Uqcrfs1, Uqcrb*, and *Uqcr10*). Thus, FoxK1 and FoxK2 upregulate and downregulate many of the nuclear-encoded components of mitochondrial oxidative phosphorylation and the cytochrome C pathway (Supplementary Fig. 6c).

To determine the impact of these changes on fatty acid β-oxidation, we analyzed the levels of fatty acid oxidation (FAO) in control and knockdown cells using palmitate/BSA as substrate (Fig. 6b). DKD cells showed over a 70% reduction in basal FAO (Fig. 6c), a 55% reduction in ATP-coupled FAO (Fig. 6d), and a 73% reduction in maximal FAO (Fig. 6e) compared with Controls. Single knockdown of FoxK1 or FoxK2 showed intermediate reductions (Fig. 6c–e). We then analyzed the mitochondrial function by measuring the oxygen consumption rate of cells using pyruvate/glucose as substrates (Fig. 6f). Again, DKD cells showed significant reductions in basal respiration (17.8 %), ATP-production (21.3%), and maximal respiration (21.0%) compared with Controls, and the single FoxK1 KD and FoxK2 KD cells showing intermediate reductions (Fig. 6g–i).

These changes in mitochondrial function were associated with altered mitochondrial morphology as visualized by electron microscopy. Both the single KD and DKD cells had mitochondria with reduced matrix density, disrupted and scanty cristae and a more rounded shape (Fig. 6j) in comparison with the control mitochondria with normal ameboid shape and well-preserved cristae (Fig. 6j). Despite the decrease in mitochondrial size, there was an increase in copy number of the mitochondrial genes *mt-Nd1* and *mt-Nd6* relative to the nuclear gene (*Gapdh*) by qPCR in FoxK1 KD and DKD cells presumably as a compensatory reaction to the mitochondrial dysregulation (Fig. 6k Supplementary Fig. 6g). The effects of FoxK1/K2 DKD on mitochondrial respiration activity and the levels of fatty acid oxidation (FAO) were rescued by re-expression of FoxK1 and FoxK2 (Fig. 6l–p and Supplementary Fig. 6h–k).

### Role of FoxK1 in cell proliferation and survival

**Fig. 7:**
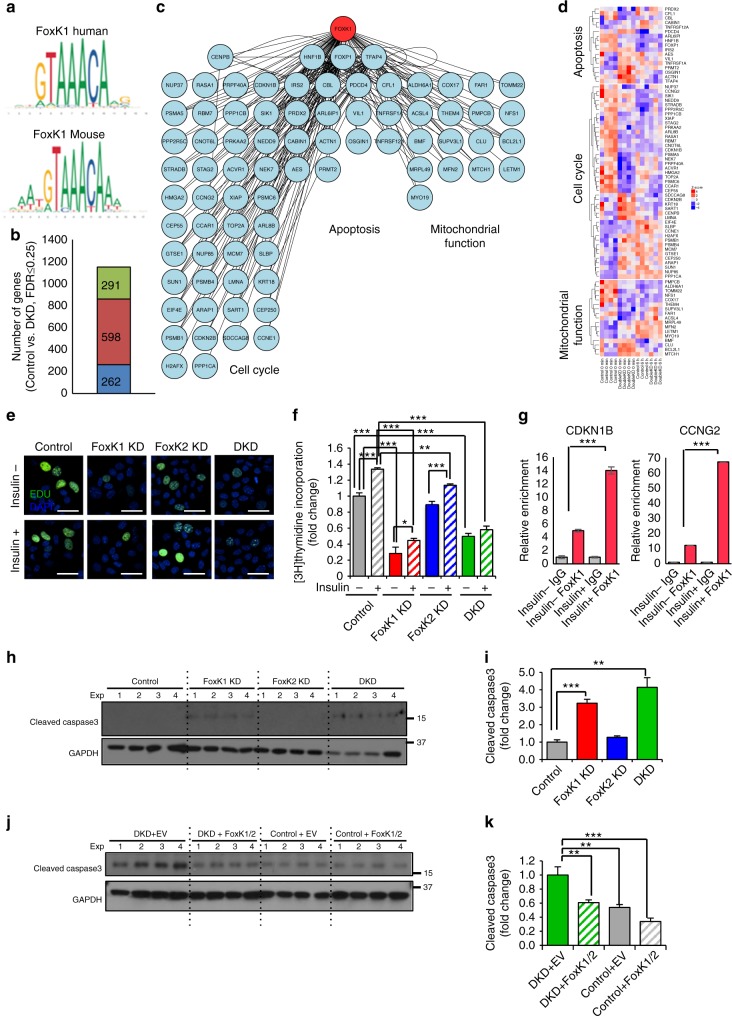
Increased apoptosis and reduced proliferation in FoxK1/K2 KD cells. **a** The promoter consensus sequences for FoxK1 in mice or humans based on Bowman, C. J. et al.^23^. **b** The bar graph shows the number of genes (262) carrying the consensus FoxK1/K2 motifs in the promoter regions among 1,151 genes that were differentially regulated in the DKD cells after insulin stimulation compared with those of the Control. **c**, **d** The network profile and the heatmap showed three groups of genes with the FoxK1/K2 motifs in the promoter regions as involved in the cell cycle, apoptosis and mitochondria-related genes that were highly affected after insulin stimulation in the Control and DKD cells. **e** Representative images of EdU (green) incorporation in Control. Foxk1 KD, Foxk2 KD and DKD AML12 cells incubated with 100 µM insulin or vehicle for 24 h. EdU is stained green, and nuclei are stained blue (DAPI). Scale bars, 50 μm. **f** [^3^H]-thymidine incorporation in Control, FoxK1 KD, FoxK2 KD and DKD AML12 cells with or without insulin stimulation for 24 h. (One-way ANOVA followed by Tukey-Kramer post hoc analysis, *P < 0.05; **P < 0.01; ***P < 0.001, *n* = 4). **g** qChip of FoxK1 at the promoter regions of indicated genes in AML12 cells before and 30 min after 100 nM insulin treatment. (One-way ANOVA followed by Dunnett’s post hoc analysis, *P < 0.05; **P < 0.01; ***P < 0.001, *n* = 4). **h** Immunoblotting for cleaved caspase3 in lysates from cells after 24 h serum starvation. **i** Densitometric analysis of cleaved caspase3 in all groups. (One-way ANOVA followed by *t*-test with Bonferroni correction, *p < 0.05; **,*p* < 0.01; ***p < 0.001, *n* = 4). **j** Immunoblotting for cleaved caspase3 in lysates from Control (NS siRNA), DKD, Control + Flag-FoxK1/K2 and DKD + Flag-tagged FoxK1/K2 after serum starvation 24 h. **k** Densitometric analysis of cleaved caspase3 in the groups shown in panel **i**. (One-way ANOVA followed by *t*-test with Bonferroni correction, *P < 0.05; **P < 0.01; ***P < 0.001, *n* = 4). All data are represented as mean ± SEM

Using a computational approach, we aligned the potentially regulated genes in the RNA-seq data with the potential promoter consensus sequences^23^ for the Forkhead/winged-helix motif of FoxK1 and FoxK2 (Fig. 7a and Supplementary Fig. 7a). Comparing the 1151 genes differentially regulated in the FoxK1/K2 DKD cells after insulin stimulation to changes in control cells (at a FDR < 0.25, |FC| > 1.5), we found 262 genes, including 13 transcription factors, which have a FoxK1 and/or FoxK2 consensus motif in their promoter regions (Fig. 7a). Another 598 genes have motifs in their promoter regions that could be targeted by the 13 FoxK1/K2-regulated transcription factors. Thus, 860 (262 + 598) of the genes whose expression was altered contain either FoxK1/K2 motifs or motifs regulated by the downstream transcription factors in their promoter regions. The remaining 291 genes do not contain any of these motifs, suggesting that they are regulated by other transcriptional events (Fig. 7b). Gene set analysis revealed that genes related to cell proliferation, apoptosis, and mitochondrial metabolism carrying the FoxK1/K2 motif were among the most markedly altered in DKD cells (Fig. 7c, d and Supplementary Fig. 7b).

Given that DKD cells showed dysregulation of genes involved in cell proliferation in response to insulin stimulation, we examined the role of FoxK1 and FoxK2 on cell growth. Both FoxK1 KD and the DKD cells showed 52–74% decreases in [^3^H]-thymidine and 5-ethynyl-20-deoxyuridine (EdU) incorporation into DNA after insulin stimulation compared with control cells, indicating the important role of FoxK1 on insulin stimulation of DNA synthesis and mitogenesis (Fig. 7e, f). Consistent with a role of FoxK1 in cell proliferation, chromatin immunoprecipitation (ChIP), and quantitative PCR (qChIP) analysis showed that FoxK1 can interact directly with the promoter regions of Cyclin-dependent kinase inhibitor 1B (*Cdkn1b)* and Cyclin-G2 (*Ccng2)*, both of which carry a FoxK1 consensus motif, following insulin stimulation (Fig. 7g). We also observed differences in the basal proliferation rates of FoxK1 KD cells compared with control cells (Fig. 7e, f) and differences in the basal of levels of expression of genes involved in cell cycle (Supplementary Fig. 7c-e). Likewise, FoxK1 KD and DKD cells showed downregulation in multiple genes involved in cell cycle regulation, while FoxK2 KD cells which showed minimal effect on proliferation altered only a small number of these genes (Supplementary Fig. 7f, g). Thus, FoxK1 KD and DKD cells have a lower proliferation potential despite the enhanced ERK1/2 phosphorylation upon insulin stimulation (Fig. 4g, h).

While FoxK1 KD and FoxK2 KD cells caused some changes in expression in common downstream targets, they showed clear differences in both upregulated and downregulated genes in the pathway analysis (Supplementary Fig. 7h). Thus, FoxK1 KD resulted in decreases in genes in pathways associated with cell cycle, cell survival, biosynthetic and metabolic, and stress response, while FoxK2 KD showed decreases in a small number of pathways, including genes involved in secretion and bile acid metabolism. Likewise, FoxK1 KD caused increases in expression of genes associated with extracellular matrix, apoptosis and intracellular calcium influx, while FoxK2 KD caused increases in expression of genes associated with cell–cell interactions and immune responses. Consistent with the altered expression of genes involved in cell survival and apoptosis in FoxK1 KD cells, both FoxK1 KD or DKD cells showed significantly increased caspase 3 cleavage compared with control cells following prolonged serum starvation (Fig. 7h, i). Re-expression of exogenous FoxK1 and FoxK2 reversed the levels of cleaved caspase 3 in the DKD cells to those of the control (Fig. 7j, k) indicating an anti-apoptotic effect of FoxK1 and FoxK2.

## Discussion

Insulin and IGF-1 are potent regulators of cell growth and metabolism. These effects are mediated by changes in protein phosphorylation, protein localization and activity, and gene expression. Despite considerable progress over the past decade in understanding the molecular mechanisms underlying these effects, except for members of the FoxO family, most other potential regulators of the transcriptional effects of these hormones remain poorly understood. The present study provides important insights into this process by showing that in addition to FoxO transcription factors, insulin and IGF1 receptors also regulate the function of FoxK family of Forkhead factors, specifically FoxK1 and FoxK2. This regulation involves multisite phosphorylation via the actions of Akt, GSK3, and mTOR, leading to translocation of FoxK1/K2 from the cytoplasm to the nucleus and the nuclear chromatin fraction, i.e., in the opposite direction of FoxOs. Using mutational analysis, we show that GSK3 phosphorylation at S402/S406 and S454/S458 in FoxK1 play a major role in its translocation. Thus, under basal conditions, FoxK1/K2 are localized in the cytoplasm, and this is dependent on GSK3-mediated phosphorylation. After insulin stimulation, there is activation of Akt with increased phosphorylation of GSK3. This results in a decrease in GSK3 activity, decreased phosphorylation of FoxK1/K2 at this serine phosphorylation motif and translocation of FoxK1/K2 into the nucleus where they can both activate and repress gene expression^25^. This is associated with a change in transcriptional profile decreasing mitochondrial oxidation and favoring long-term survival of cells during active cell proliferation.

There are multiple levels of crosstalk between insulin signaling and FoxK1/K2. First, these transcription factors can be co-immunoprecipitated with these receptors in a ligand-dependent manner. Second, translocation of FoxK1/K2 occurs co-incident with translocation of phosphorylated IR/IGF1R to the nucleus. However, we could not find any evidence of direct Tyr phosphorylation of FoxK1 by the receptor. Rather, as noted above, this translocation is dependent on serine phosphorylation of FoxK1, which is downstream of Akt, mTOR, and GSK3. Although the exact molecular mechanism of translocation remains to be determined, the translocation of FoxK1 coincident with phosphorylated IRβ into nucleus and toward chromatin may depend on interaction with yet another protein involved in this complex.

In addition, there is a feedback loop such that FoxK1/K2 play a regulatory role in insulin signal transduction by modifying the signaling through Akt and ERK, such that knockdown of FoxK1/K2 result in enhanced Akt/ERK phosphorylation/activation. Transcriptome analysis supports the role of FoxK1/K2 as a modifier of insulin-mediated signal transduction pathways. Thus, in FoxK DKO cells insulin has lost its ability to up-regulate multiple genes involved in regulation of signal transduction including the dual specific phosphatase *Dusp5* involved in regulation of ERK^28^, chordin (*Chrd*) involved in TGFβ-signaling and BMP signaling^29^, tyrosine protein phosphatase type 5 (*PTPN5*) involved in suppression of ERK^30^, protein phosphatase 1, catalytic subunit, alpha isoform (Ppp1ca) involved in suppression of Akt^31^, myosin heavy chain 10 (*Myh10*) that is associated with AMPK and involved in insulin sensitivity^32^, neuritin 1 (*nrn1*) which is involved in upregulation of outward current (IA) subunit Kv4.2 expression and increases IA densities, in part activating IR signaling^33,34^, Sprouty homolog 4 (*SPRY4*) involved in MAPK inhibition^35^, C–C chemokine receptor (*CCR9*) involved in insulin sensitivity^36^, and methionine adenosyltransferase 1A (*Mat1a*) which is involved in triglyceride storage and insulin sensitivity^37^. Exactly how this combination of FoxK regulation of signal transduction genes alters insulin and IGF-1 action remains to be determined.

One of the most interesting aspects of FoxK1/K2 regulation is that it is reciprocal to FoxO signaling. In the fasting or unstimulated state, FoxO1 localizes predominantly in the nucleus. Insulin induces Akt activation through IRS-1 and IRS-2 leading to the phosphorylation of FoxO1 which favors its translocation into the cytoplasm and binding to 14–3–3 proteins, thus blocking its transcriptional activity. The targets of FoxOs are multiple and include genes involved in metabolism, cell proliferation, apoptosis, autophagy, inflammation, and stress resistance^38^. This is analogous to the regulation of the Forkhead transcription factor Daf-16 in *C. elegans*^39^. FoxOs and Daf-16 bind to two response elements in DNA, the Daf-16 member binding element (5′-GTAAA(C/T)AA-3′) and the insulin-response element (5′-(C/A)(A/C)AAA(C/T)AA-3′)^40^. Previous studies^23^ and our current work suggest that FoxKs bind to a similar motif [(A/T)(G/A)TAAA(C/T)A]. However, we have identified this motif in the promoters of only about 25% of genes regulated in the FoxK1/K2 DKD cells, suggesting that some of the changes in gene expression are regulated by transcription factors downstream FoxK1/K2. In addition to binding directly to DNA, FoxK proteins also may be recruited to chromatin through protein–protein interactions similar to FoxO proteins.

In mammals, FoxO1 has been intensively studied as one of the key regulatory molecules for glucose metabolism in liver. FoxO1 has effects on genes involved in glucose production and utilization and has little effect on genes directly involved lipid or glucose oxidation^41^. Thus, in liver, FoxO1 interacts with Pgc1α^17^ and upregulates the expression of gluconeogenic enzymes, such as G6pc and PEPCK^42^. In preadipocytes, FoxO1 increases expression of p21 and suppresses PPARγ inhibiting their differentiation to adipocytes in vitro^19,43^. FoxO1 also reduces the expression of G1-phase cyclins D1 and D2, and G2-M phase cyclin B^44^. FoxO1 phosphorylation is also influenced by acetylation and deacetylation by the NAD-dependent deacetylases Sirt1 and Sirt2^45^. Sirt1 binds directly to FoxO1 through a conserved LXXL motif and catalyzes its deacetylation^46^. FoxO1 acetylation promotes its phosphorylation leading to its retention in the cytoplasm and reduces FoxO1 transcriptional activity^47^. Insulin also regulates gene expression in liver through effects on sterol regulatory element binding protein-1c (SREBP-1c)^48^, and SREBP-1c activation is also regulated at a transcriptional level via FoxO1 and Sp1^49^.

While much remains to be learned about the physiological impact of FoxK1/K2 regulation of gene expression, FoxK1 has been shown to regulate the expression of c-myc and the p21 genes in C2C12 muscle cells^50,51^. FoxK1 also cross-talks with Wnt/β-catenin signaling by translocating Disheveled (DVL) into the nucleus^52^. In this study, we find that FoxK1/K2 regulate sets of genes involved in mitochondrial metabolism, cell apoptosis, and cell survival in AML12 liver cells. We also found that FoxK1 directly interacts with the promoter regions of cyclin-dependent kinase inhibitor 1B (*Cdkn1b)* and cyclin-G2 (*Ccng2)* following the insulin stimulation. As a result, knockdown of FoxK1 and FoxK2 causes the impairment of cell proliferation, increases cleaved caspase 3 indicating increased apoptosis, reduced expression of genes involved in mitochondrial lipid oxidation and decreased basal and maximal lipid oxidation. In addition, re-expression of FoxK1/K2 in the knockdown cells results in recovery of mitochondrial oxidative phosphorylation. Thus, FoxK1 can play a complementary role with FoxO1 in regulation of liver cell metabolism.

**Fig. 8:**
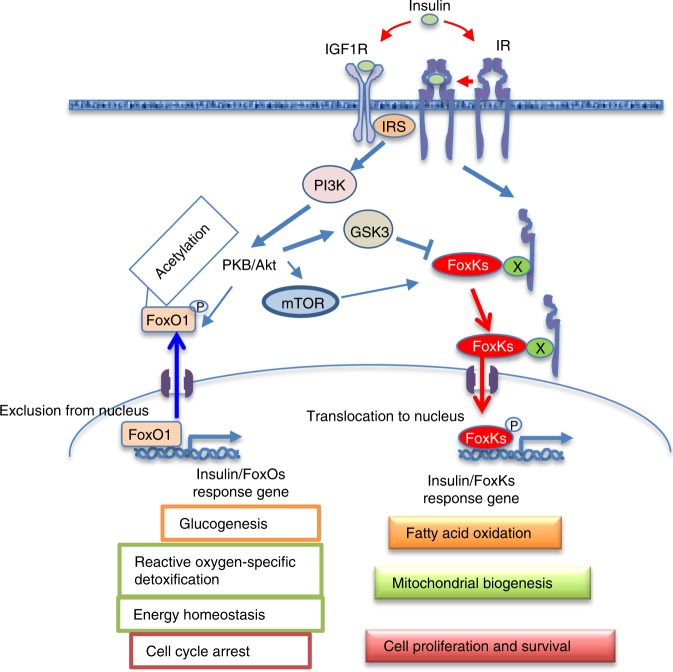
Schematic model of insulin regulation of FoxK1/K2 and FoxOs in cellular function. The FoxK Forkhead transcription factors translocate from the cytoplasm to nucleus reciprocally to the translocation of FoxO1. FoxK translocation to the nucleus is dependent on the Akt-mTOR pathway, while its localization to the cytoplasm in the basal state is dependent on GSK3. Once in the nucleus, FoxKs play important roles in regulation of genes, fatty acid oxidation, mitochondrial biogenesis, cell proliferation and survival. Where other unknown proteins (named here X) are in the FoxK and IR/IGF1R protein-protein complexes remains to be determined

Taken together, our data support a expanded model of insulin action and the role of Forkhead box transcription factors (summarized in Fig. 8). In this model, insulin signaling triggers insulin receptor substrate phosphorylation leading to PI3K-Akt activation resulting in phosphorylation and downregulation of FoxO activity by excluding FoxO from the nucleus and retaining it in the cytoplasm. Simultaneously, insulin signaling modified the phosphorylation at multiple sites resulting in the reciprocal translocation of FoxK transcription factors from cytoplasm to nucleus where they migrate to the chromatin fraction. This translocation of FoxK1/K2 is dependent on the Akt, mTOR, and GSK3 pathways, and is important in control of fatty acid oxidation, mitochondrial biogenesis, cell proliferation, and survival. The reciprocal translocation of FoxKs and FoxO1 between the cytoplasm and nucleus after insulin stimulation creates an elaborate balance in insulin regulation of metabolism, cell growth, and cell survival.

## Methods

### Materials

Antibodies against phospho-IR/IGF1R (#3024, 1:1000), IRβ (#3025, 1:1000), IGF1Rβ (#3027, 1:1000), phospho-ERK1/2 (T202/Y204) (#9101, 1:1000), ERK1/2 (#9102, 1:1000), phospho-Akt (S473) (#9271, 1:1000), Akt (#4685, 1:1000), phospho-S6 (S235/236) (#2211, 1:2000), S6 (#2317, 1:1000), phosopho-FoxO1/3 (#9464,1:1000), FoxO1 (#2880,1:1000), FoxK1(#12025,1:1000), FoxK2 (#12008,1:1000), Lamin A/C (#2032,1:1000), HistoneH3 (#4499,1:1000), GSK3α (#4337, 1:1000), GSK3β (#9315, 1:1000), Vimentin (#5741, 1:1000), Na, K/ATPase (#3010, 1:1000) were purchased from Cell Signaling Technologies. Phospho-IRS-1 (Y612) (09-432, 1:1000) antibody was purchased from Millipore. GAPDH (sc-25778, 1:1000) antibody was from Santa Cruz. Flag (F3040, 1:5000) antibody was from Sigma. Anti-IRS-1 (611394, 1:500) antibody was from BD Biosciences. For immunocytochemistry FoxK1 (ab18196) was purchased from Abcam. Human insulin was purchased from Sigma and human IGF-1 from Preprotech. Mouse 3XFlag-FoxK1 and 3XFlag-FoxK2 cDNA clones were purchased from Genecopoeia. Human HA-GSK3βcDNA clone was from Addgene. Mouse IR (MC224356) and IGF1R (MC224342) cDNA clones were from Origene. Wild-type IR and IGF1R, as well as chimeric receptors IR/IGF1R [IR extracellular domain (a.a. 1–919) fused to IGF1R transmembrane and intracellular domain (a.a. 908–1339), numbers excluding signal peptide] and IGF1R/IR [IGF1R extracellular domain (a.a. 1–907) fused to IR transmembrane and intracellular domain (a.a. 920–1345)] were subcloned into the pBabe-hyrgromycin vector^22^. To generate chimeric receptors, an Ile^947^ to Leu point mutation was introduced into the insulin receptor cDNA to generate a BclI restriction site using the primer pair (5′-ccatcaaatattgccaaactgatcattggacccctcatc-3′; IR Bcll 3: 5′-gatgaggggtccaatgatcagtttggcaatatttgatgg-3′).

### Mice

Mice were maintained on a 12-h light/dark cycle and fed a normal CD (9F5020; PharmaServ). Tissues from 3-month-old male C57BL/6J mice were collected and rinsed in PBS. To assess insulin signaling in vivo, 5 U insulin (Sigma-Aldrich) was injected via the inferior vena cava. *FoxK1*^*lox/lox*^ mice were created using a targeting construct with loxP sites flanking exons 4 and 5 of *FoxK1*. These mice were injected via the tail vein with adenovirus encoding Cre recombinase gene to induce recombination in the liver or adenovirus encoding GFP gene as a control. All animal studies followed the National Institutes of Health guidelines and were approved by the Institutional Animal Care and Use Committees at the Joslin Diabetes Center or University of Gothenburg.

### Brown preadipocytes isolation and culture

Preadipocytes were isolated from newborn IR-lox/IGF1R-lox mice by collagenase digestion of brown fat and immortalized by infection with retrovirus encoding SV40 T-antigen followed by the selection with 2 µg ml^−1^ of puromycin. The immortalized preadipocytes were infected with adenovirus containing GFP alone (to generate control cell line) or GFP-tagged Cre recombinase (to generate IR/IGF1R double knockout preadipocytes). GFP-positive cells were sorted and expanded in DMEM supplemented with 10% heat-inactivated fetal bovine serum (FBS, Sigma), 100 U/ml penicillin and 100 µg/ml streptomycin (Gibco) at 37 °C in a 5% CO_2_ incubator^2^. IR/IGF1R double knockout preadipocytes were then stably transduced using the pBabe retrovirus system to generate mouse 6XHis-tagged IR, IGF1R, IR/IGF1R IGF1R/IR chimeric receptor cell lines^22^. Briefly, human embryonic kidney 293T cells (ATCC) were transiently transfected with 10 µg of the pBabe-hygro retroviral expression vectors encoding wild-type or mutant IR or IGF1R sequences and viral packaging vectors SV-E-MLV-env and SV-E-MLV using TransITExpress transfection reagent (Mirus Bio Corp.). Forty-eight hours after transfection, virus-containing medium was collected and passed through a 0.45 µm syringe filter. Polybrene (hexadimethrine bromide; 12 µg/ml) was added, and the medium was applied to proliferating (40% confluency) DKO cells. Twenty-four hours after infection, cells were treated with trypsin and re-plated in a medium supplemented with hygromycin (Invitrogen). Cells were maintained in DMEM supplemented with 10% FBS, 100 U/ml penicillin and 100 µg/ml streptomycin (Gibco), and cultured at 37 °C in a humidified atmosphere of 5% CO_2_.

### Insulin and IGF-1 signaling and subcellular fractionation

Cells were serum starved for 3 h with DMEM containing 0.1% BSA. Cells expressing wild-type IR, WT IGF1R, chimeric receptor IR/IGF1R or IGF1R/IR were stimulated with 10 or 100 nM insulin for indicated times, while cells expressing wild-type IGF1R or chimeric receptor IGF1R/IR were stimulated with 10 or 100 nM IGF-1 matching the extracellular domain for indicated times. After stimulation, cells were washed immediately with ice-cold PBS once before lysis and scraped down in RIPA buffer (Millipore, 20–188) supplemented with phosphatase inhibitor and protease inhibitor cocktail (Sigma-Aldrich) and 1× phosphatase inhibitor cocktail (Sigma-Aldrich). Subcellular protein fractionation was performed using the subcellular protein fractionation kit (Thermo Scientific Pierce) according to the manufacturer’s instructions. Protein concentrations were determined using the Pierce 660 nm Protein Assay Reagent (Bio-Rad). Lysates (10 to 20 µg) were resolved on SDS-PAGE gels, transferred to PVDF membrane for immunoblotting.

### Immunoblotting

Membranes were blocked in Starting Block T20 (ThermoFisher) at room temperature for 1 h, incubated with the indicated primary antibody in Starting Block T20 solution overnight at 4 °C. Membranes were washed three times with 1 × PBST, incubated with HRP-conjugated secondary antibody (1:10,000) in Starting Block T20 for 1 h and signals were detected using Immobilon Western Chemiluminescent HRP Substrate (Millipore). All uncropped immunoblotting images are presented in Supplementary Figs. 8–14.

### Chemical cross-linking and pull-down assay

For the chemical cross-linking, the cells were washed with PBS three times and incubated for 30 min at 4 °C with 1 mM DTSSP (Thermo Fisher) in PBS, followed by washing three times with TBS (20 mM Tris-HCl, 100 mM NaCl, pH 7.4) before use in the following procedure. To examine protein interactions, cells expressing His-tagged normal IR, His-tagged normal IGF1R, His-tagged chimeric receptor IR/IGF1R or IGF1R/IR were lysed in lysis buffer [20 mM Hepes (pH 7.4), 150 mM NaCl, 50 mM KF, 50 mM β-glycerolphosphate, 2 mM EGTA (pH 8.0), 1 mM Na_3_VO_4_, 1% Triton X-100 or 1% NP-40, 10% glycerol, 1× protease inhibitor cocktail (Sigma) and 5 mM imidazole] and then centrifuged for 20 min at 21,200 ×*g*. Fifteen milligram protein lysates were incubated with Talon Metal Affinity Resin (100 μl beads equilibrated in Lysis buffer), (Clontech/Takara, 635501) in a total volume of 14 ml for 1 h at 4 °C with end-to-end rotation. The protein complex-bound resin was washed three times with Lysis buffer and eluted the bound His-tagged protein with Elution buffer [20 mM Hepes (pH 7.4), 150 mM NaCl, 50 mM KF, 50 mM β-glycerolphosphate, 2 mM EGTA (pH8.0), 1 mM Na_3_VO_4_, 1% Triton X-100 or 1% NP-40, 10% glycerol, 1× protease inhibitor cocktail (Sigma) and 200 mM imidazole] by end-to-end rotation at 4 °C for 5 min.

### Liquid chromatography tandem mass spectrometry (LC-MS/MS)

Sample processing steps included SDS-PAGE purification of proteins, in-gel protein digestion using trypsin and peptide labeling with TMT 10-plex reagents. Multiplexed quantitative mass spectrometry data were collected on an Orbitrap Fusion mass spectrometer operating in a MS^3^ mode using synchronous precursor selection for MS^2^ fragment ion selection^53^. MS^2^ peptide sequence data were searched against a Uniprot mouse database with both the forward and reverse sequences using the SEQUEST algorithm. Further data processing steps included controlling peptide and protein level false discovery rates, assembling protein groups, and protein quantification from peptides.

### Cell lines and transient siRNA and plasmid transfections

Cells were seeded at 1 × 10^5^ cells cm^−2^ for 24 h prior to the transfection. For siRNA treatment, cells were transfected with 8 μl RNAiMax per 1.3 ml medium and 25 or 50 nM siRNA according to the manufacturer’s protocol. The target sequences for siRNA treatment are provided in Supplementary Table 7. For transfection of plasmids, cells were transfected with 4 μl of Lipofectamine 3000 and 2.5 μg DNA each per 1.3 ml medium. The medium was changed on the next day.

### Insulin stimulation for RNA isolation

Cells were serum starved overnight with DMEM + 0.1% BSA then mock treated or treated with insulin (100 nM) for 6 h, after which they were washed once with cold 1× PBS and resuspended in RLT lysis buffer (Qiagen). Total RNA was extracted using an RNeasy mini kit (Qiagen) following manufacturer’s manual.

### RNA-Seq processing and data analysis

RNA-Seq analysis was performed by the BioPolymers Facility at Harvard Medical School. Library for RNA-Seq was prepared using NEBNext mRNA Sample Prep Master Mix kit (NEB), and sequencing was performed using an Illumina HiSeq2500 platform in the Biopolymer core facility at Harvard Medical School. Statistical significance of transcripts was assessed with empirical Bayesian linear modeling using the *limma* package^54^, and significance of gene sets was assessed with the *sigPathway* package^55^. Heatmaps were created with the *gplots* package, and volcano plots and scatterplots were created with the *ggplot2* package^56^.

### qRT-PCR and mitochondrial DNA copy number

One microgram of RNA was reverse transcribed using a High Capacity cDNA Reverse Transcription kit (Applied Biosystems) according to the manufacturer’s instructions. Real-time PCR was performed using the SYBR Green PCR master mix (Bio-Rad). Fluorescence was monitored and analyzed in an ABI Prism 7900 HT sequence detection system (Applied Biosystems). TBP expression was used to normalize gene expression. Amplification of specific transcripts was confirmed by analyzing melting curve profiles at the end of each PCR. The mitochondrial DNA (mtDNA) copy number was measured by comparing mt-ND1 and mt-ND6 (mtDNA) to GAPDH (nuclear DNA). All primer sequences can be found in Supplementary Table 7.

### Immunofluorescence

Cells grown on glass coverslips were fixed with 4% formaldehyde for 15 min at room temperature, rinsed three times in PBS plus 0.3% Triton for 5 min and blocked in 5% BSA for 30 min at room temperature. Cells were then incubated with anti-FoxK1 antibody (Abcam ab18196) and secondary antibody coupled to AlexaFluor 488 (Life Technologies). Cells were mounted on glass slides using Vectorshield hard set mounting medium (Vector Laboratories). Images were acquired with a two-photon confocal microscope (Zeiss 710). For EdU incorporation assay, cells were treated with 10 μM EdU (8 h) and stained with Click-iT Plus EdU Alexa Fluor 488 Imaging Kit (Thermo Fisher).

### Cell proliferation assay using [^3^H]-thymidine incorporation

Cells were plated 5 × 10^4^ per well to 24-well plates. On the next day cells were starved with serum-free DMEM/F12 containing 0.1% BSA media for 24 h then labeled with 0.2 μCi [^3^H]-thymidine per well for 24 h in the presence or absence of 100 μM insulin. After labeling, the cells were washed twice with PBS and precipitated with 500 μl of 10% TCA for 10 min at −20 ℃. [^3^H]-thymidine incorporation was quantified by scintillation counter.

### Seahorse bioanalyzer

A Seahorse XFe96 Flux analyzer (Agilent Technologies) was utilized to measure Oxygen Consumption Rate (OCR) according to manufacturer’s protocol. Cells were seeded into XFe 96 cell culture microplates at the density of 20,000 cells per well. One day before cells were incubated with substrate-limited medium (DMEM, 0.5 mM glucose, 1 mM GlutaMAX, 0.5 mM carnitine, 1% FBS) to prime cells for fatty acid utilization. One hour before the assay, cells were given 2.5 mM glucose and 0.5 mM carnitine in the running medium (111 mM NaCl, 4.7 mM KCl, 1.25 mM CaCl_2_, 2 mM MgSO_4_, 1.2 mM NaH_2_PO_4_ and 5 mM HEPES), To determine the Fatty Acid Oxidation (FAO), FAO running media was added with the 0.175 mM palmitate-BSA FAO substrate (Agilent Technologies) followed by addition of 1 µM oligomycin (oligo) from, 0.5 μM FCCP, and 2 µM antimycin (Ant). Prior to oligomycin injection, basal FAO was calculated with the subtraction of non-mitochondrial respiration by the addiction of the carnitine palmitoyltransferase-1 (CPT1) inhibitor 40 mM etomoxir. After 1 µM oligomycin injection, ATP-coupled FAO was calculated. And the maximal FAO is calculated from Maximal FCCP rate minus non-mitochondrial respiration. For the quantifications in the bar graphs, data points for all wells across the four time-points were averaged. To measure parameters of mitochondrial function, the XF Cell Mito Stress Test was utilized by directly measuring the OCR of cells. In this experiment, cells were given pyruvate and 25 mM glucose in the running medium followed by sequential addition of 1 µM oligomycin, 1 µM FCCP, and 2 µM antimycin. Basal respiration, ATP production, and maximal respiration capacity was calculated with the subtraction of non-mitochondrial respiration. For the quantitation in the bar graphs, data points for all wells across the four time-points were averaged. Cells were lysed in 0.1% SDS solution and protein concentrations were measured and used for normalization of OCR values.

### Transmission electron microscopy

Cells were fixed in 0.1 M sodium phosphate buffer containing 2.5% glutaraldehyde for 16 h and post-fixed with 2% osmium tetroxide in Millonig Buffer, then processed in a standard manner and embedded in BEEM capsules filled with Araldite resin. Semi-thin sections were cut at 1 μm and stained with 1% toluidine blue to evaluate the quality of preservation. Ultrathin sections (60–80 nm) were cut, mounted on copper grids and stained with uranyl acetate and lead citrate by standard methods. Stained grids were examined and photographed on a Philips 301 transmission electron microscope using a side mount Infinity 2 digital camera with Infinity Camera V:3.1 software from Lumenera Scientific.

### Chromatin immunoprecipitation (ChIP) assay

Cells were first stimulated with insulin or saline and then cross-linked for 10 min by adding formaldehyde directly to tissue culture medium to a final concentration of 1%. Cross-linked cells were then washed twice with cold PBS, scraped, pelleted, resuspended in Chip lysis buffer (0.5% NP40, 85 mM KCl, 10 mM HEPES (pH 8.0), 5 mM DTT, PMSF and 1× protease inhibitor cocktail), and incubated for 10 min on ice and spun for 10 min. The supernatant was discarded. The cell pellet was incubated in Nuclei lysis buffer (1% SDS, 10 mM EDTA, 50 mM Tris-HCL pH 8.0, 1× protease inhibitor cocktail) for 10 min on ice then sonicated to produce chromatin fragments of 300–1000 bp. The samples were centrifuged, and the supernatants diluted in dilution buffer with protease inhibitors and precleared with 50 μl protein A/G magnetic beads for 1 h at 4 °C. Cross-linked chromatin was incubated 3 h with 10 μg FoxK1 antibody or control IgG at 4 °C. Antibody-protein-DNA complexes were isolated by immunoprecipitation with 50 μl protein A/G magnetic beads. After extensive washing, immune complexes were eluted using freshly prepared elution buffer (50 mM Tris-Cl (pH 8.0), 10 mM EDTA, 1% SDS). Formaldehyde cross-linking was reversed by overnight incubation at 65 °C. Samples were purified by standard phenol:chloroform extraction and used as a template in PCR. The primers used were as follows: Cdkn1B forward, 5’-GGCCGTTTGGCTAGTTTGTT-3′ and reverse, 5′-CTGGTCGCGTGACTACTCG-3′; Ccng2 forward, 5′-TACTTTGGGCGGACTTTTCA-3′ and reverse, 5′-GCGGAAGGAGACAGTTTCTG-3′; and reverse, 5′-GCGGAAGGAGACAGTTTCTG-3′. Samples from at least three independent immunoprecipitations were analyzed.

### Phosphoproteome sample preparation and LC-MS/MS

All samples were lysed in SDS lysis buffer (4% SDS, 10 mM DTT, 10 mM HEPES pH 8), boiled and sonicated, and precipitated overnight using ice-cold acetone (v/v = 80%). After centrifugation at 4000×*g*, the pellet was washed twice with 80% ice-cold acetone before air drying and resuspended with sonication in TFE buffer (10% 2-2-2-trifluorethanol, 100 mM ammonium bicarbonate (ABC)). Proteins were digested using LysC and trypsin (1:100), over-night at 37 °C. The following day, phosphopeptides were enriched as follows^26^. Peptides were loaded on a 50 cm reversed phase column (75 µm inner diameter, packed in-house with ReproSil-Pur C18-AQ 1.9 µm resin [Dr. Maisch GmbH]). Column temperature was maintained at 60 °C using a homemade column oven. An EASY-nLC 1200 system (Thermo Fisher Scientific) was directly coupled online with the mass spectrometer (Q Exactive HF) via a nano-electrospray source, and peptides were separated with a binary buffer system of buffer A (0.1% formic acid (FA)) and buffer B (80% acetonitrile plus 0.1% FA), at a flow rate of 350 nl min^−1^. Peptides were eluted with a nonlinear 150-min gradient of 5–60% buffer B (0.1% (v/v) formic acid, 80% (v/v) acetonitrile). After each gradient, the column was washed with 95% buffer B for 5 min. The mass spectrometer was programmed to acquire in a data-dependent mode (Top10) using a fixed ion injection time strategy. Full scans were acquired in the Orbitrap mass analyzer with resolution 60,000 at 200 *m*/*z* (3E6 ions were accumulated with a maximum injection time of 10 ms). The top intensity ions (N for TopN) with charge states ≥2 were sequentially isolated to a target value of 1E5 (maximum injection time of 120 ms, 20% underfill), fragmented by HCD (NCE 27%) and detected in the Orbitrap (*R* = 15,000 at *m*/*z* 200).

### Data processing and analysis

Raw mass spectrometry data were processed using MaxQuant version 1.5.3.15^57,58^ with an FDR < 0.01 at the level of proteins, peptides, and modifications. Searches were performed against the Mouse UniProt FASTA database (September 2015). Enzyme specificity was set to trypsin. The search included cysteine carbamidomethylation as a fixed modification and N-acetylation of protein, oxidation of methionine, and phosphorylation of Ser, Thr, Tyr residue (PhosphoSTY) as variable modifications. Upto 2 missed cleavages were allowed for protease digestion. ‘Match between runs’ was enabled, with a matching time window of 0.7 min. Bioinformatic analyses and data visualization were performed with Perseus (www.perseus-framework.org)^59^. Significance was assessed using ANOVA analysis, for which replicates were grouped, and statistical tests performed with permutation-based FDR correction for multiple hypothesis testing. Missing data points were replaced by data imputation after filtering for valid values (all valid values in at least one experimental group).

### Statistics

All data are presented as mean ± SEM and analyzed by two-tailed Student’s *t* test or one-way ANOVA followed by post hoc comparisons as appropriate. N indicates the number of animals per group or number of independent experiments. Results were considered significant if *P* < 0.05.

## Supplementary information

Supplementary Information

## Data Availability

RNA-seq data generated in this study are available at NCBI GEO database with the accession number GSE110574 and can be accessed with the following access link: [https://www.ncbi.nlm.nih.gov/geo/query/acc.cgi?acc=GSE110574]. Proteomic data are available at Github and can be accessed with the following access link: [https://github.com/jdreyf/Foxk12-insulin]. All the other data in this study are available from the corresponding author on reasonable request.
