# Supplementary material for: FoxK1 and FoxK2 in insulin regulation of cellular and mitochondrial metabolism
Source: Nat Commun. 2019 Apr 5;10:1582. doi: 10.1038/s41467-019-09418-0 (PMC6450906; doi:10.1038/s41467-019-09418-0)
Supplement: Supplementary file 1 — Supplementary Information [file 41467_2019_9418_MOESM1_ESM.pdf]

**Supplementary Information**

# FoxK1 and FoxK2 are Components in Insulin Regulation of Cellular and Mitochondrial Metabolism

Sakaguchi et al

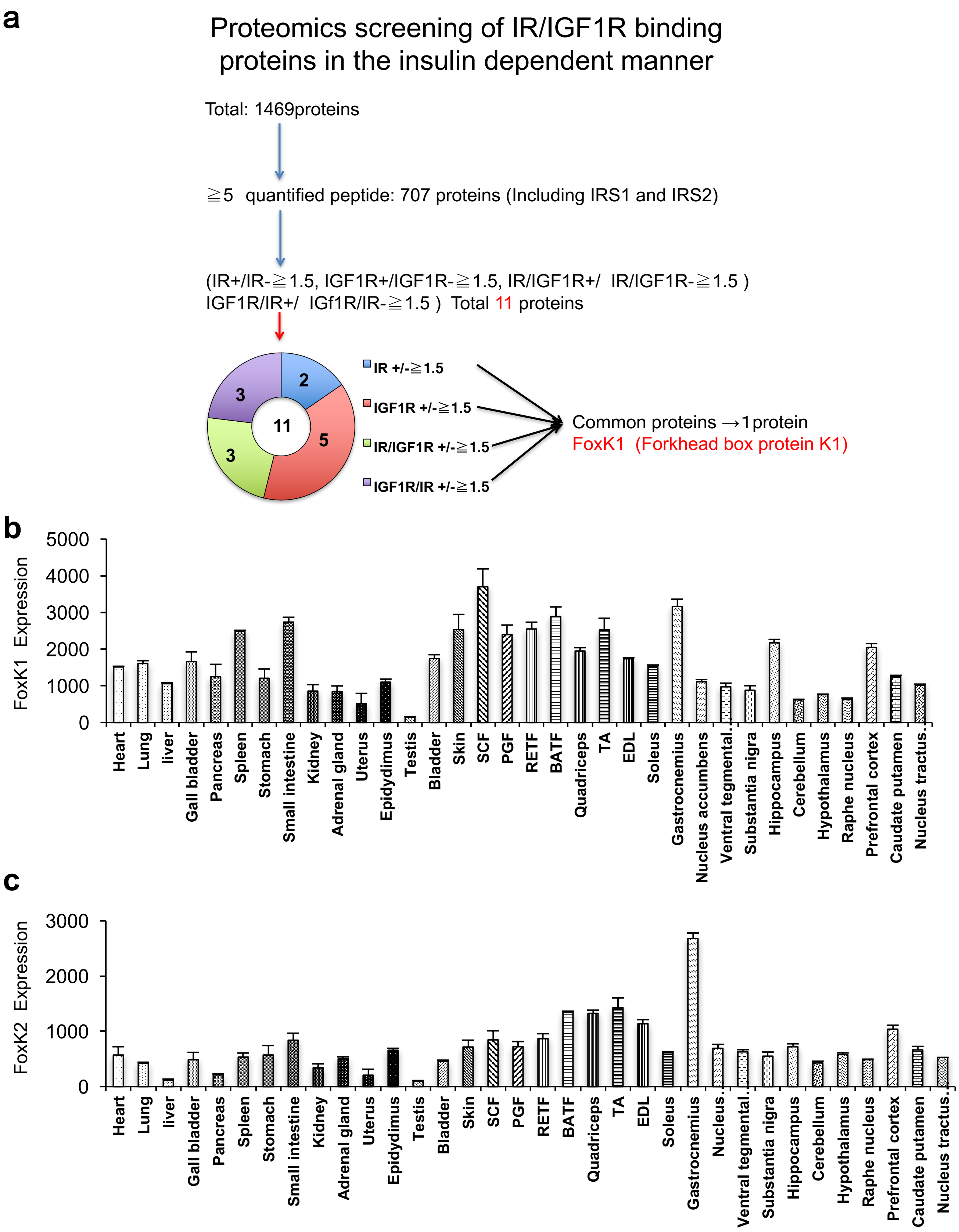


**Sakaguchi et al., Supplementary Figure 1**

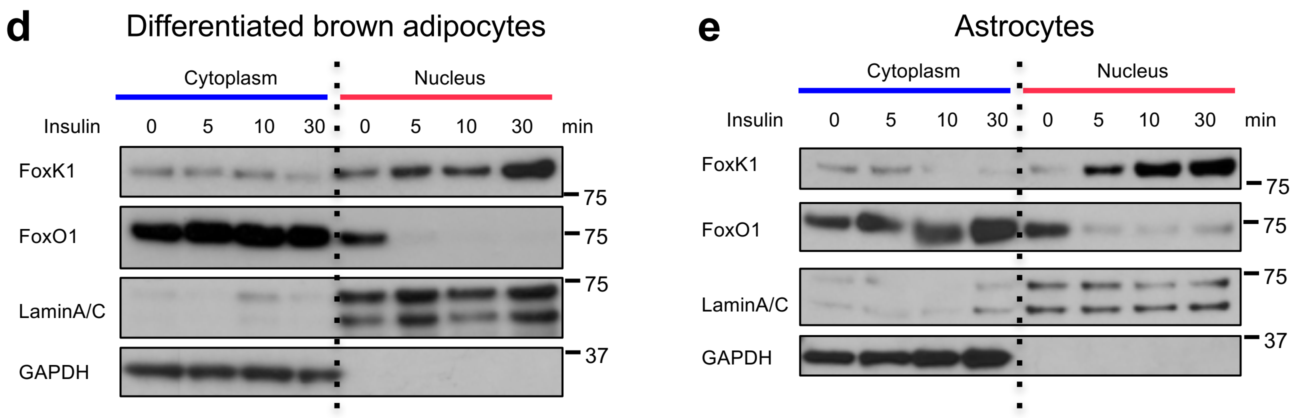


**Supplementary Figure 1. FoxK1 is a novel component of IR- and IGFR-mediated signaling complex.**
(**a**) FoxK1 is identified by the proteomics screening of IR/IGF1R binding proteins. (**b**) FoxK1 and (**c**) FoxK2 expression (qPCR) from 34 tissues from 3-month-old C57BL/6J mice (All data are represented as mean ± SEM, n=3). Data were normalized to *Tbp*. Immunoblotting of FoxO1 and FoxK1 in nuclear and cytoplasmic fractions extracted from differentiated brown preadipocytes **(d)** or astrocytes **(e)** before and after 100 nM insulin stimulation for indicated times.

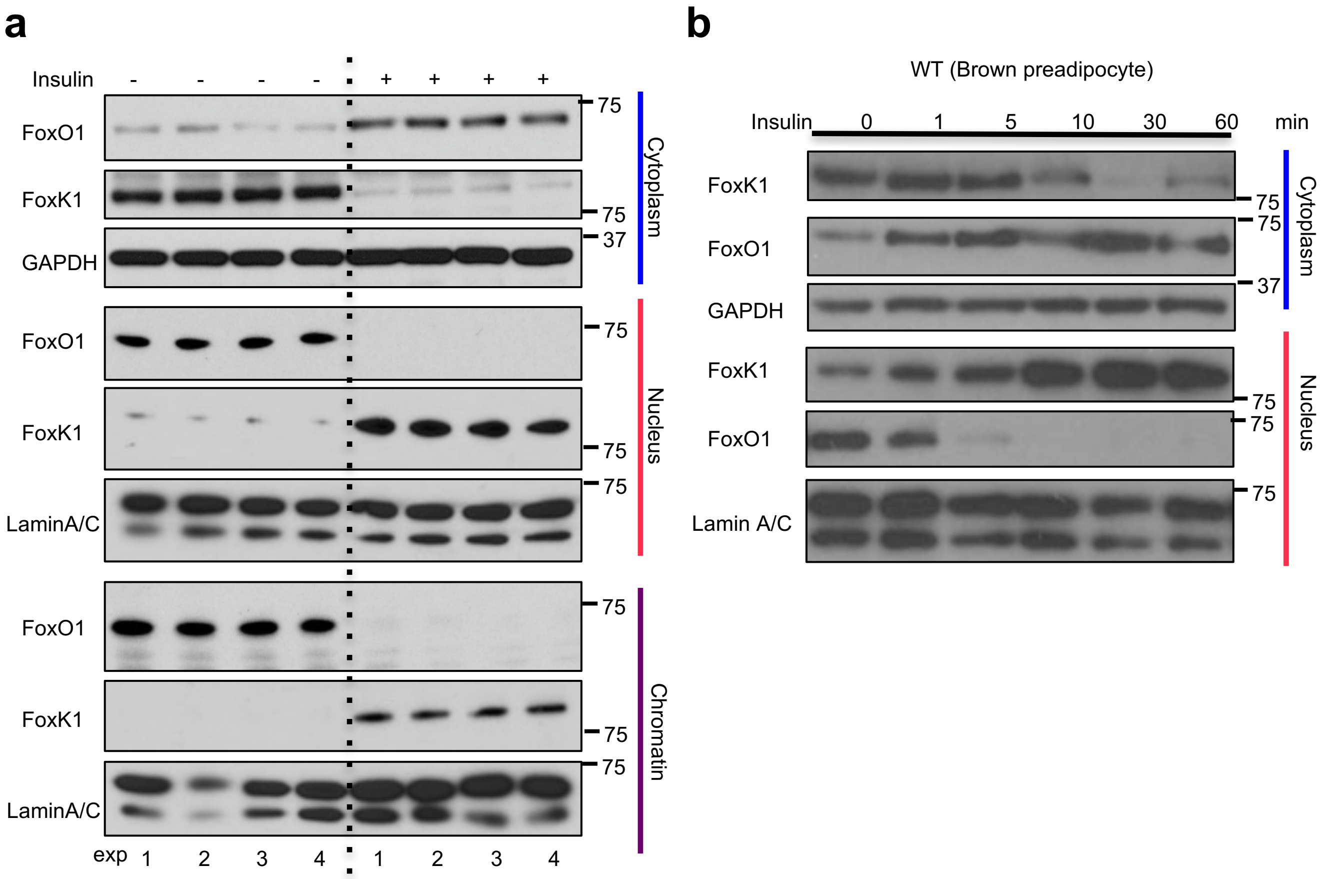


**Supplementary Figure 2. Insulin-induced FoxK1 translocation is reciprocal to FoxO1. (a)** Subcellular fractions of cytoplasm, nucleus and chromatin were prepared from DKO brown preadipocytes reexpressing the IR without (0 min) and with 100 nM insulin stimulation for 30 min. FoxO1 and FoxK1 in each fraction were assessed by western blotting, as were markers for different fractions, cytosol (GAPDH), nuclear and chromatin (Lamin A/C) (n = 4). **(b)** Immunoblotting of FoxO1 and FoxK1 in nuclear and cytoplasmic fractions extracted from wild-type brown preadipocytes before and after 100 nM insulin stimulation for 30 min.

**Sakaguchi et al., Supplementary Figure 2**

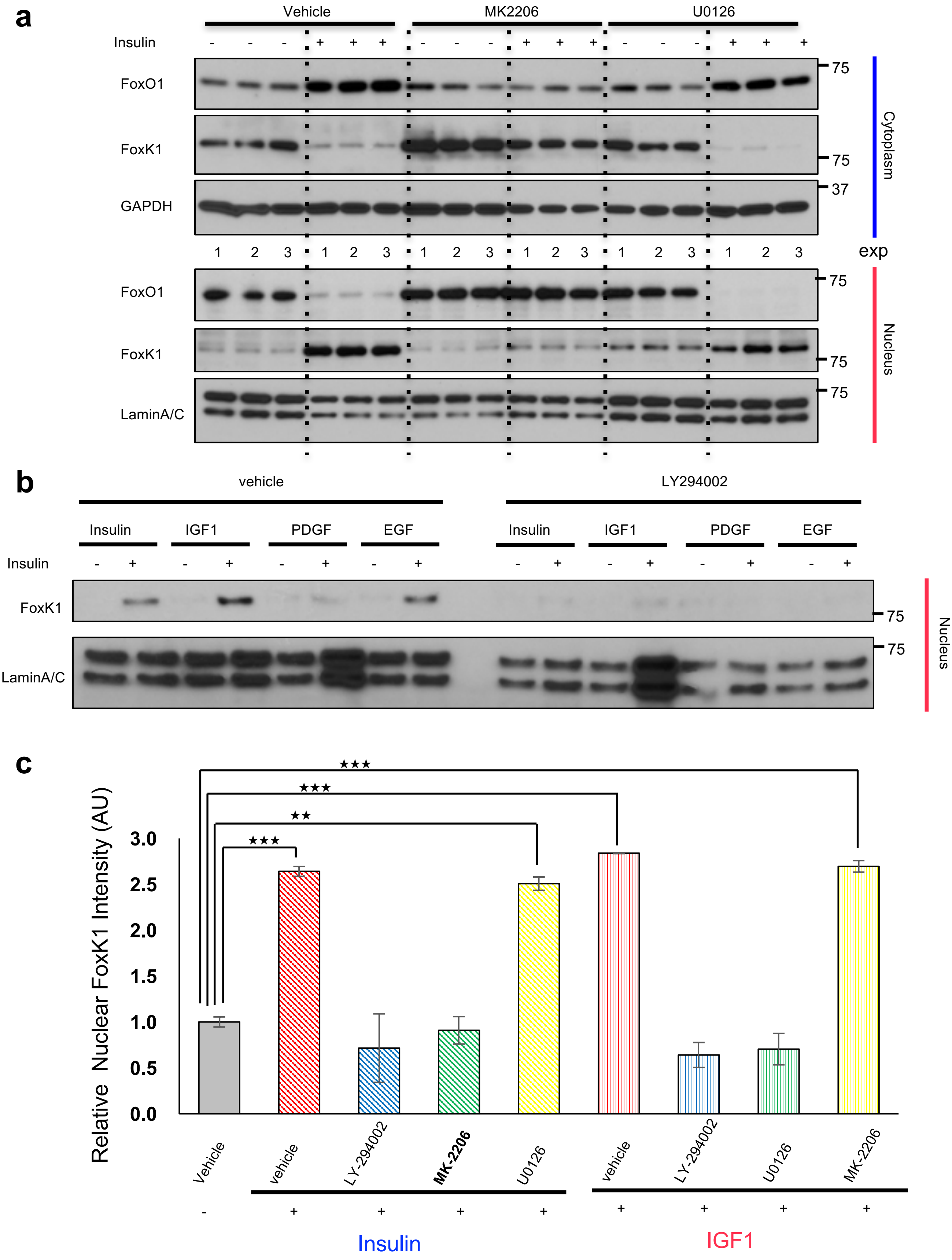


**Sakaguchi et al., Supplementary Figure 3**

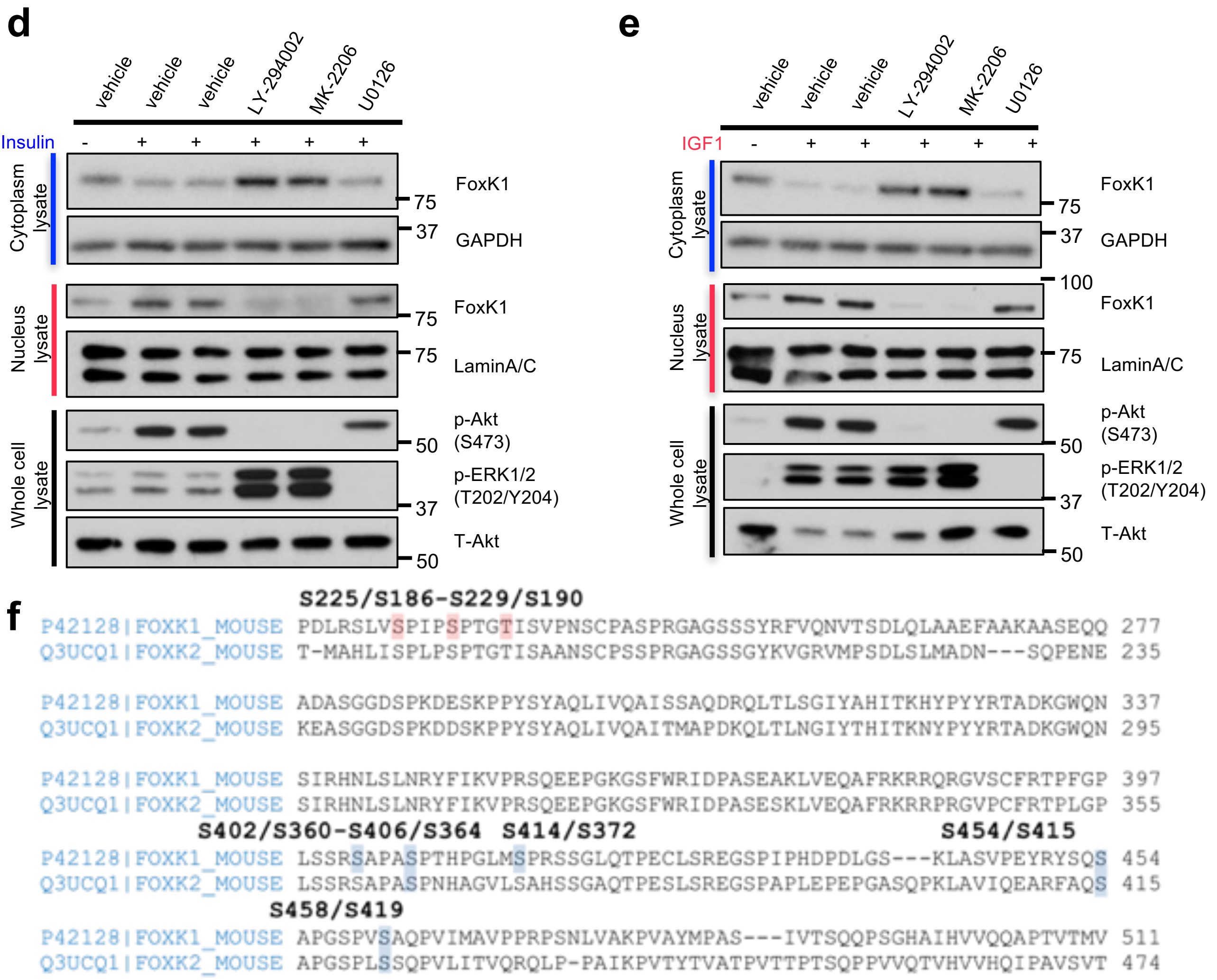


**Supplementary Figure 3. Insulin-induced FoxK1 translocation is regulated by PI3K-Akt rather than ERK MAPK pathway. (a)** Immunoblotting of FoxO1 and FoxK1 in nuclear and cytoplasmic fractions extracted from IR-expressing brown preadipocytes before and after 10 nM insulin stimulation for 30 min in the presence or absence of the Akt inhibitor MK2206 (5 μM) or the MEK1/2 inhibitor U0126 (20 μM) (n = 4). **(b)** Immunoblotting of FoxK1 in nuclear fraction extracted from WT-brown preadipocytes before and after stimulation with insulin, IGF1, PDGF and EGF at 15 min in the presence or absence of the PI3K inhibitor (LY-294002). (**c**) Relative FoxK1 fluorescence intensity in nuclear (DAPI$^{+}$) area quantified by Image J in Figure 2d. All data are represented as mean ± SEM. ($P < 0.05$; **, $P < 0.01$; ***, $P < 0.001$) (n = 4). **(d,e)** Immunoblotting of FoxO1 and FoxK1 in nuclear and cytoplasmic fractions extracted from AML12 cells before or after stimulation with 100 nM insulin and IGF1 for 30 min in the presence or absence of the Akt inhibitor MK2206 (5 μM) or the MEK1/2 inhibitor U0126 (20 μM). **(e)** Comparison of the phosphorylation clusters between FoxK1 and FoxK2 after Insulin/IGF1 stimulation. The significant increase after Insulin/IGF1 stimulation shown in red and the decrease shown in blue.

**Sakaguchi et al., Supplementary Figure 3**

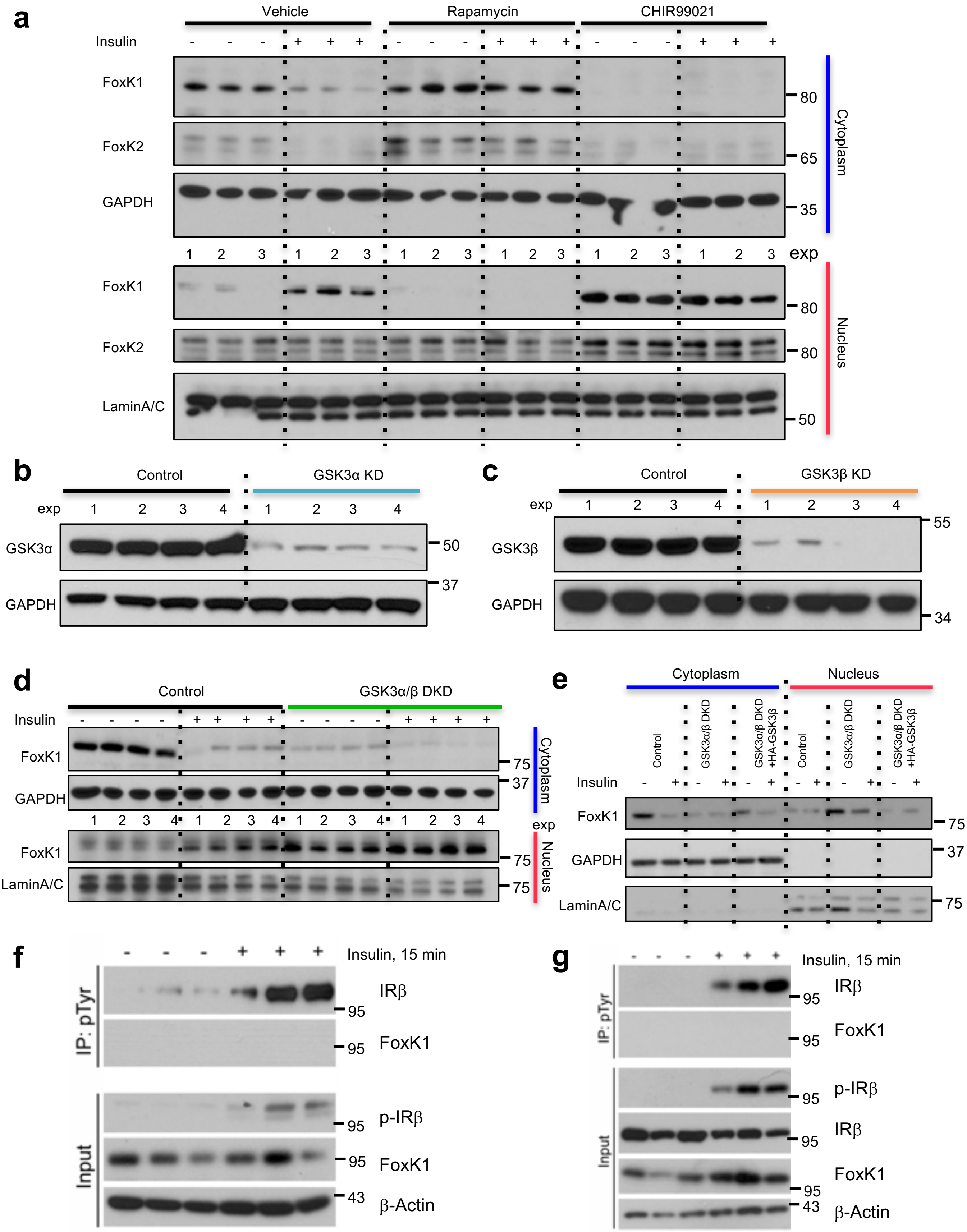


Sakaguchi et al., Supplementary Figure 4

h

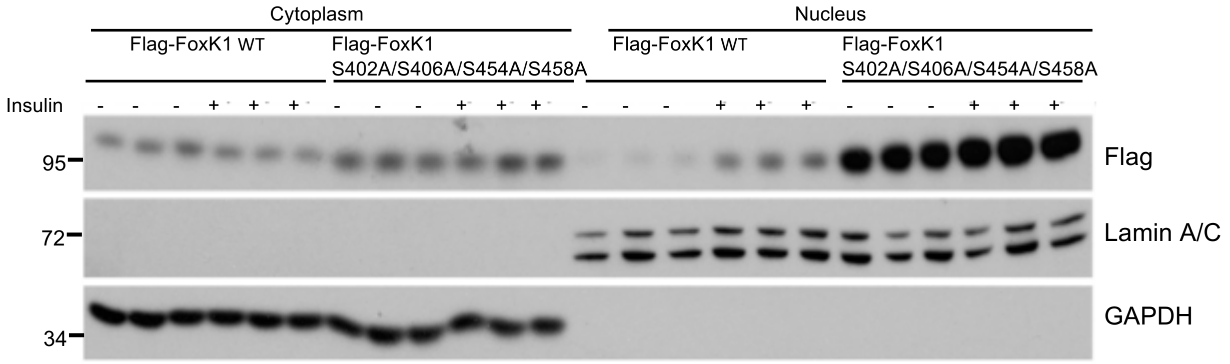


**Supplementary Figure 4. Insulin-induced FoxK1 translocation is regulated by not only mTOR but also GSK signaling. (a)** Nuclear cytoplasmic fractionation and immunoblotting for FoxK1 and FoxK2 extracted from the AML12 cells before and after 100 nM insulin at 30 min the presence or absence of 100 nM rapamycin (mTOR inhibitor) or/and 10 μM CHIR99201 (GSK3 inhibitor). GAPDH is cytosolic marker and Lamin A/C is nuclear marker (n = 3). Immunoblotting Control (NS siRNA), GSK3α-knockdown (GSK3α KD) **(b)** and GSK3β-knockdown (GSK3β KD) **(c)** cells by siRNAs with anti-GSK3α and GSK3β antibodies (n = 4). **(d)** Nuclear and cytoplasmic fractionation and immunoblotting of FoxK1 extracted from the Control (NS siRNA) or GSK3α/β double knockdown cells before (0 min) and after 100 nM insulin stimulation for 30 min. (n = 4). (**e**) Nuclear and cytoplasmic fractionation and immunoblotting of FoxK1 extracted from the Control (NS siRNA) , GSK3α/β DKD cells and re-expressed with HA-GSK3β in GSK3α/β DKD cells. before (0 min) and after 100 nM insulin stimulation for 30 min.(**f**) Immunoblotting with anti-IRβ, p-IRβ and FoxK1 antibody in lysates extracted from AML12 cells which were immunoprecipitated with p-Tyr antibody following insulin stimulation. (**g**) Immunoblotting with anti-IRβ, p-IRβ and FoxK1 antibody in lysates extracted from liver samples which were immunoprecipitated with p-Tyr antibody following insulin stimulation via vena cava injections. (**h**) Nuclear and cytoplasmic fractionation and immunoblotting of Flag extracted from the overexpressed 3XFlag-FoxK1 wild type (WT) cells or overexpressed 3XFlag-FoxK1 S402A/S406A/S454A/S458A mutant cells before (0 min) and after 100 nM insulin stimulation for 30 min.

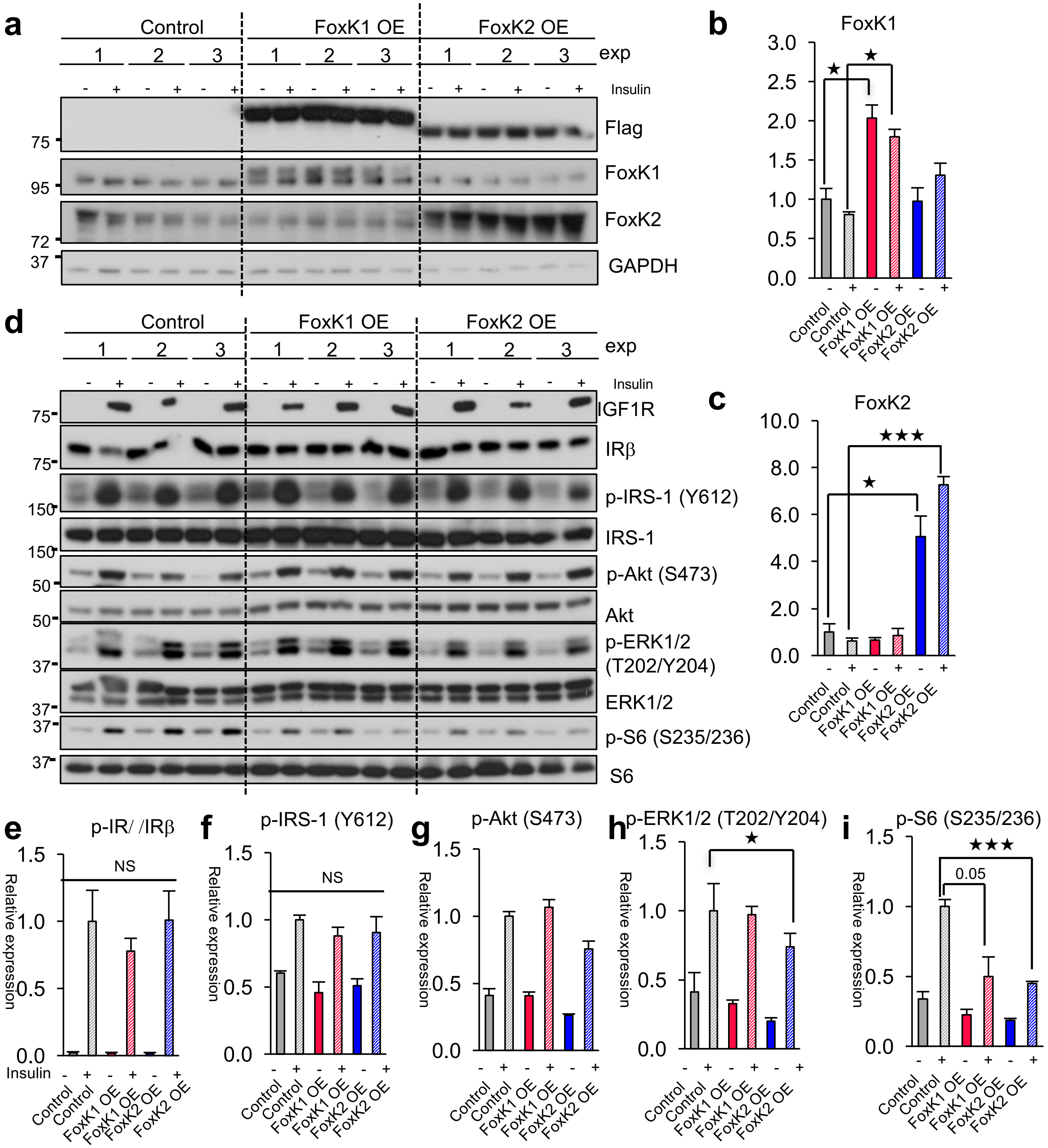


**Supplementary Figure 5. Regulation of insulin-mediated signal transduction in overexpression of FoxK1 or FoxK2 cells.** **(a)** Immunoblotting for Flag, FoxK1 and FoxK2 in lysates from Control (Mock) AML12 cells or cells overexpressed either 3XFlag-FoxK1 (FoxK1 OE) or 3XFlag-FoxK2 (FoxK2 OE) with 100 nM insulin for 5 min. **(b-c)** Densitometric analysis of phosphorylated FoxK1 and FoxK2 following 5 min stimulation. Data are mean ± SEM of n = 3 (One-way ANOVA followed by *t*-test with Bonferroni correction , *, $p < 0.05$; **, $p < 0.01$; *** $p < 0.001$. ). **(d)** Immunoblotting of the phosphorylation of IR, IRS-1, Akt, ERK, and S6 in lysates from Control (Mock) AML12 cells or cells overexpressed either 3XFlag-FoxK1 (FoxK1 OE) or 3XFlag-FoxK2 (FoxK2 OE) and stimulated with 100 nM insulin for 5 min. **(e-i)** Densitometric analysis of phosphorylated IR, IRS1, Akt, ERK, and S6 following 5 min insulin stimulation. Data are mean ± SEM of n = 3 (One-way ANOVA followed by *t*-test with Bonferroni correction , *, $p < 0.05$; **, $p < 0.01$; *** $p < 0.001$).

**Sakaguchi et al., Supplementary Figure 5**

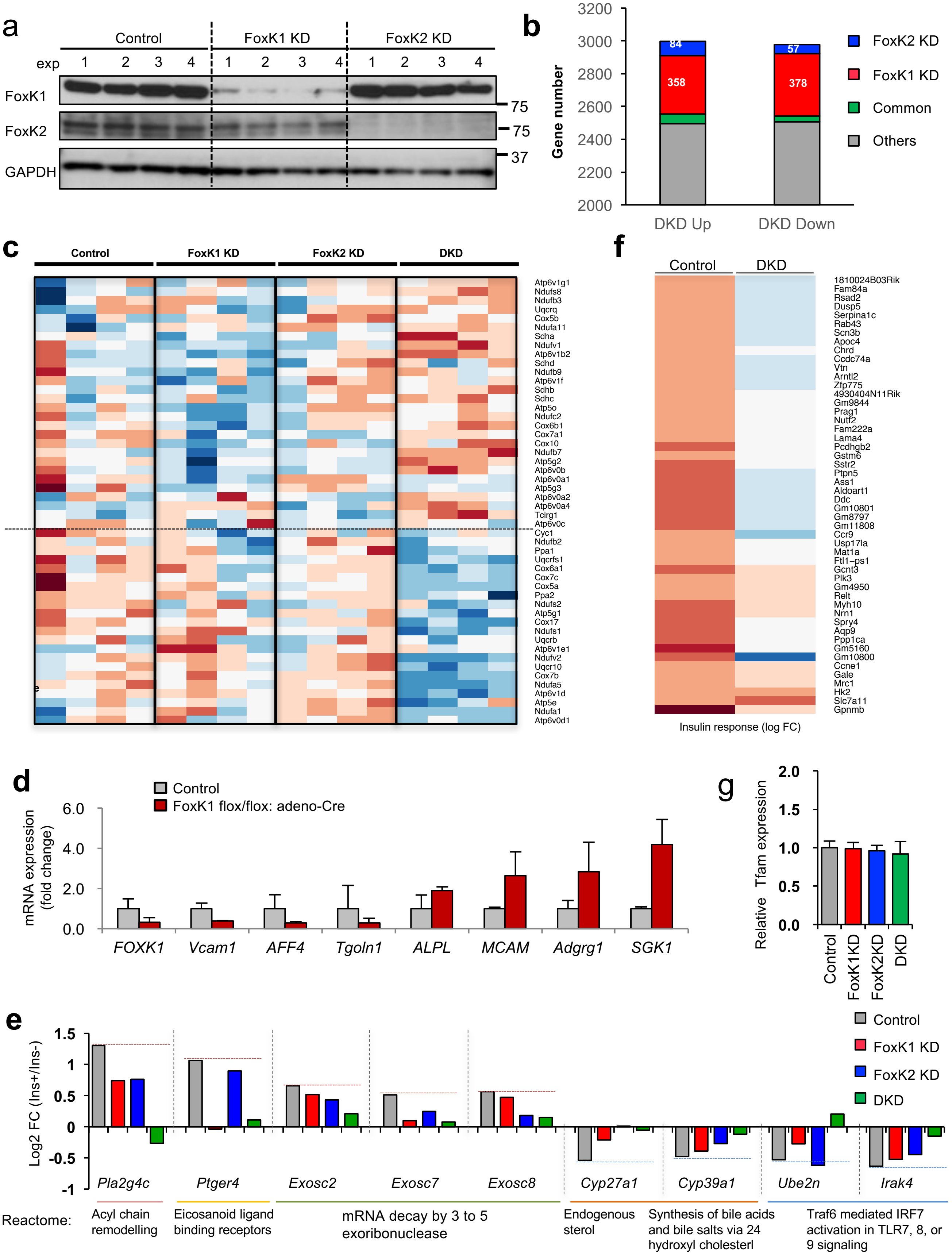


**Sakaguchi et al., Supplementary Figure 6**

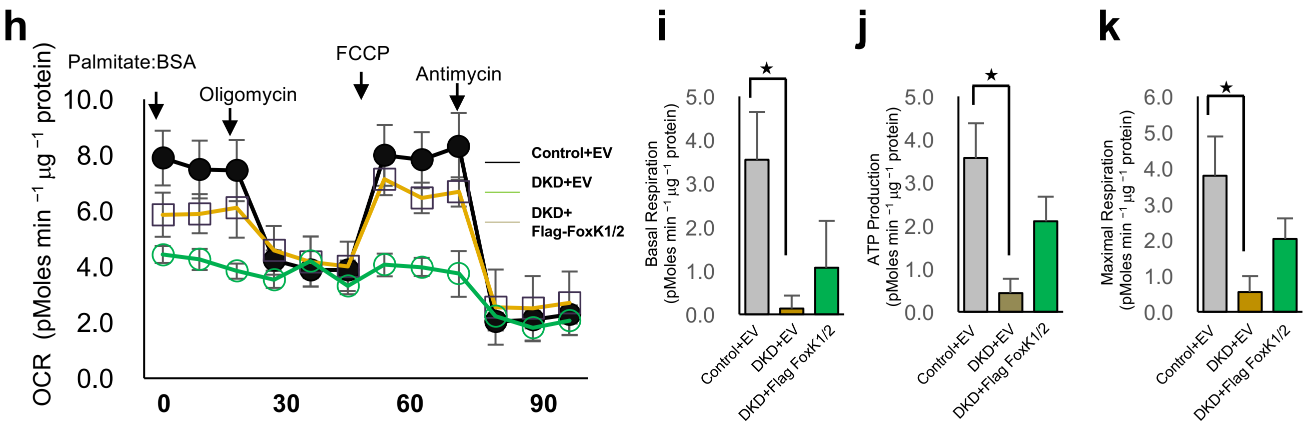


**Supplementary Figure 6. Differential gene regulation by loss of FoxK1 and FoxK2.** **(a)** Immunoblotting Control (NS siRNA), FoxK1 knockdown (FoxK1 KD) and FoxK2 knockdown (FoxK2 KD) cells by siRNAs with anti-FoxK1 and FoxK2 antibodies. (n = 4). (**b**) Among the 2996 genes, 358 (12%) genes were selectively upregulated and 378 (12.6%) genes were selectively downregulated in FoxK1 KD cells, whereas only 84 (3%) genes were selectively upregulated and 57 (2%) genes were selectively downregulated in FoxK2 KD cells. **(c)** Heat map of mitochondrial oxidative phosphorylation genes in all groups. (**d**) mRNA abundance measured by real-time qPCR in liver from Contol (FoxK1$^{flox/flox}$: adeno-GFP) *n* = 4 and FoxK1KO (FoxK1$^{flox/flox}$: adeno-Cre) mice. All data are represented as mean ± SEM. **(e)** Heatmap of top 50 significant changed insulin response genes differentially regulated by Control and DKD (SHOWN only up in Control). (**f**) Alteration of reactome transcripts in KD cells compared with Controls (gray). Fold change is shown as insulin +/insulin -. (**g**) mRNA abundance measured by real-time qPCR from Control ,FoxK1KD,FoxK2KD and DKD cells *n* = 4/group. All data are represented as mean ± SEM. (**h**) Measurement of fatty acid oxidation (FAO) using Seahorse Bioanalyzer. 0.175 mM palmitate:BSA was used as substrate. One day before cells were incubated with substrate-limited medium (DMEM, 0.5 mM glucose, 1 mM GlutaMAX, 0.5 mM carnitine, 1% FBS) to prime cells for fatty acid utilization. One hour before the assay, cells were given 2.5 mM glucose and 0.5 mM carnitine in the running medium (111 mM NaCl, 4.7 mM KCl, 1.25 mM $CaCl_2$, 2 mM $MgSO_4$, 1.2 mM $NaH_2PO_4$ and 5 mM HEPES), followed by addition of 0.175 mM palmitate-BSA (pal), 1μM oligomycin (oligo) from, 0.5 mM FCCP, and 2 μM antimycin (Ant). All data are represented as mean ± SEM. **(i-k)** Quantitation of basal (**i**), ATP-coupled (**j**) and maximal (**k**) oxygen consumption rates (OCRs) of fatty acid oxidation (FAO). For the quantifications in the bar graphs, data points for all wells were averaged, and error bars represent SEM. (One-way ANOVA followed by *t*-test with Bonferroni correction , *, *P* < 0.05; **, *P* < 0.01; ***, *P* < 0.001, n = 5/group).

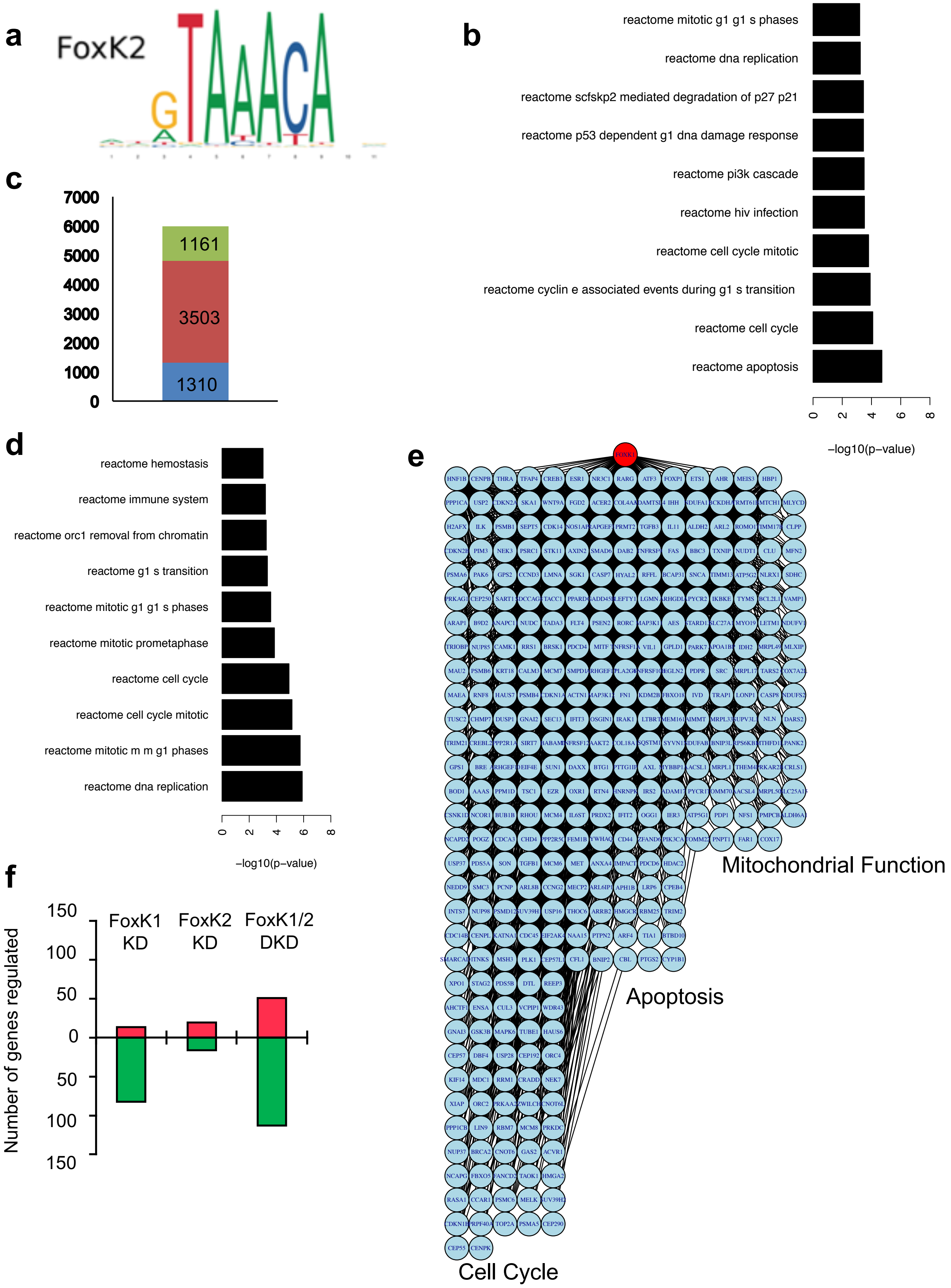


**Sakaguchi et al., Supplementary Figure 7**

g

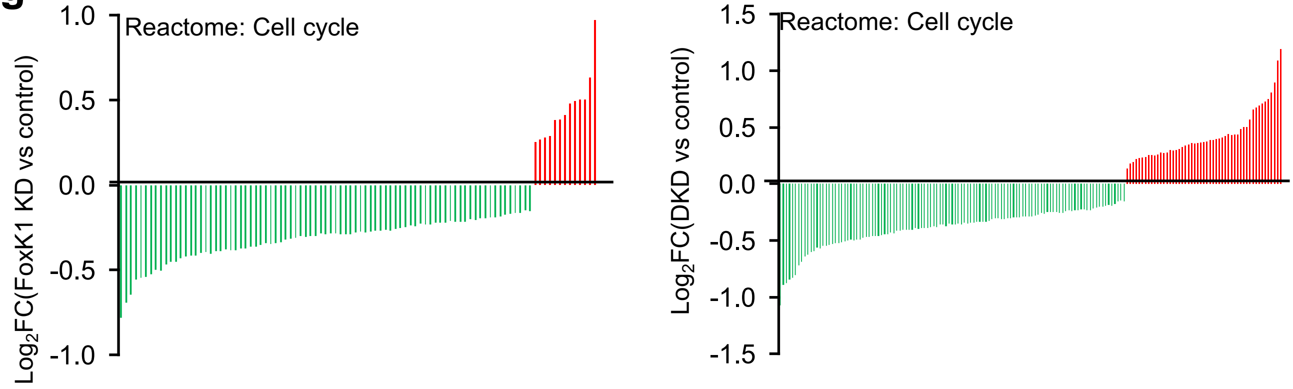


h

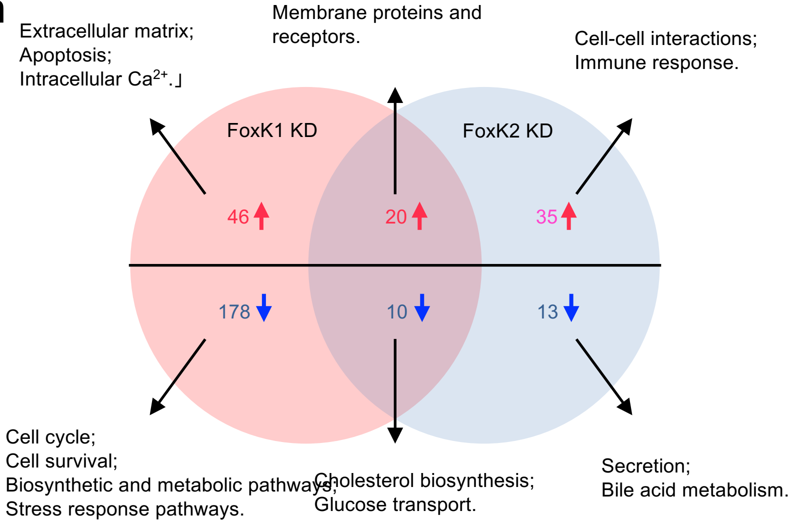


**Supplementary Figure 7. Altered target genes regulated by loss of FoxK1 and FoxK2.** (**a**) The logo shows the promoter consensus motif for FoxK2 (human) used to search the targets affected by loss of FoxK1 and FoxK2. (**b**) Top regulated pathways by insulin stimulation in Control and DKD. (**c**) The bar graph shows the number of genes (1310) including 49 transcription factors carrying the consensus FoxK1/2 motifs in the promoter regions among 5,974 genes that were differentially regulated in the DKD cells at the basal condition compared with those of the Control. Another 3,503 genes have promoter regions with the motifs that could be targeted by the 49 FoxK1-regulated transcription factors. Thus 4,813 (1,310+3,503) genes contain the FoxK1/2 motif or the motifs in the promoter regions regulated by downstram transcription factors. The remaining 1,161 genes do not contain any of these motifs, implicating that they are regulated by further downstream events. **(d)** Top differentially regulated pathways at basal condition in Control and DKD. (**e**) The network profile showed three groups of genes with the FoxK1/2 motifs in the promoter regions as involved in the cell cycle, apoptosis and mitochondria-related genes that were also highly affected at the basal condition in the Control and DKD cells. (**f**) Total number of genes within Reactome: Cell cycle that were significantly up- or down-regulated in FoxK1KD, FoxK2KD and DKD cells than control cells. ($P < 0.05$, basal conditions) (**g**) Within cell cycle pathway, the majority of the genes are significantly down-regulated in FoxK1 KD cells (left) and DKD cells (right). (**h**) Venn diagram showing the numbers of significantly regulated pathways (Reactome) in FoxK1KD and FoxK2KD cells (FDR<0.25).

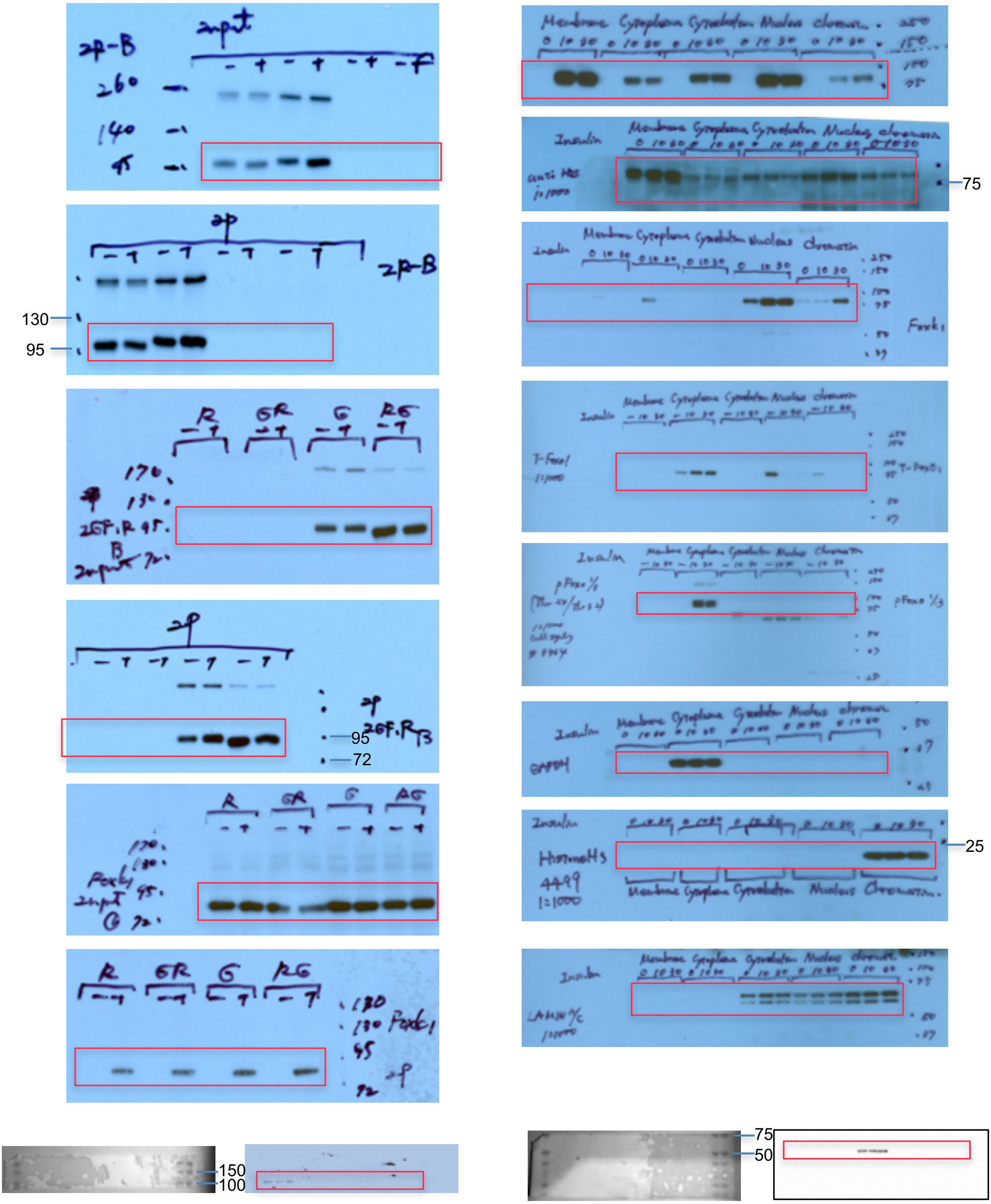


**Supplementary Figure 8. Uncropped blots.** The red sections indicate blot results shown in Figure 1.

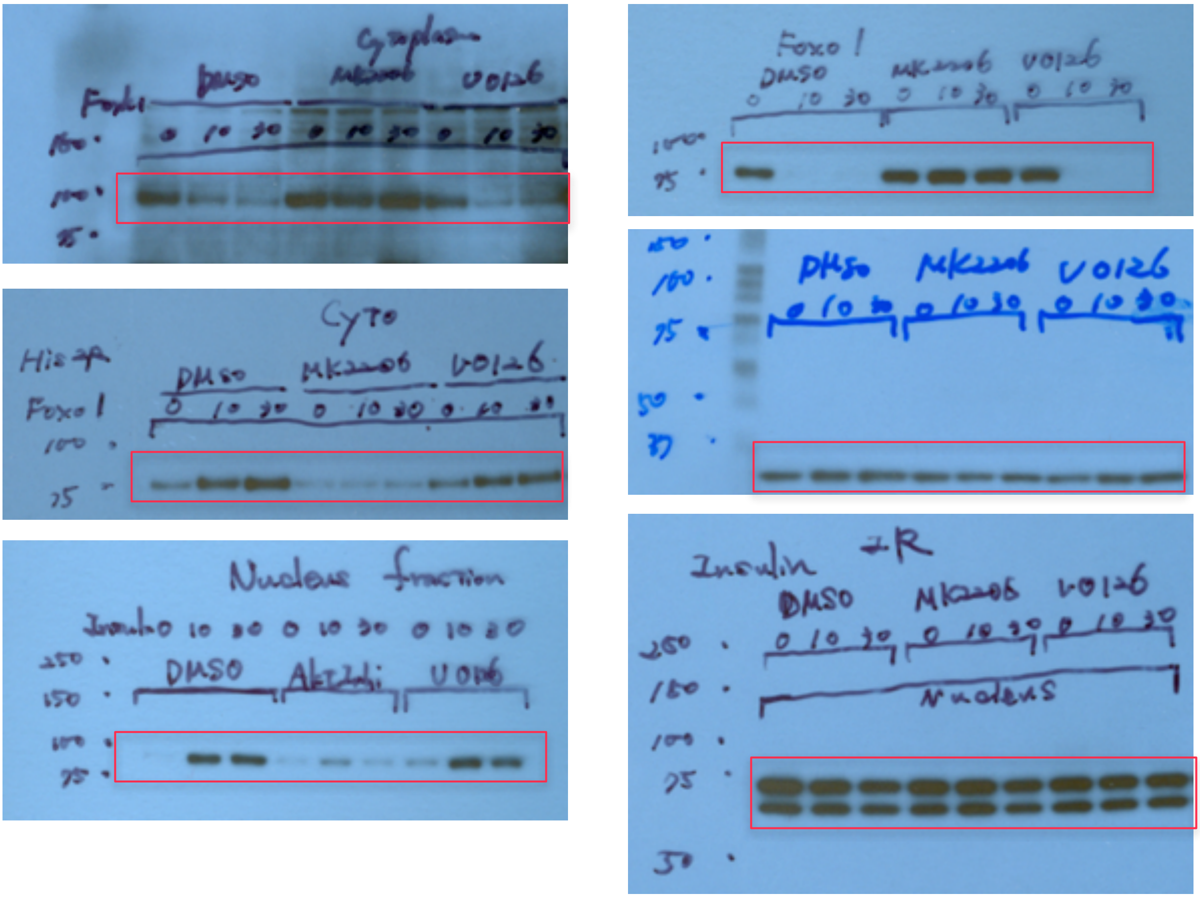


**Supplementary Figure 9. Uncropped blots.** The red sections indicate blot results shown in Figure 2.

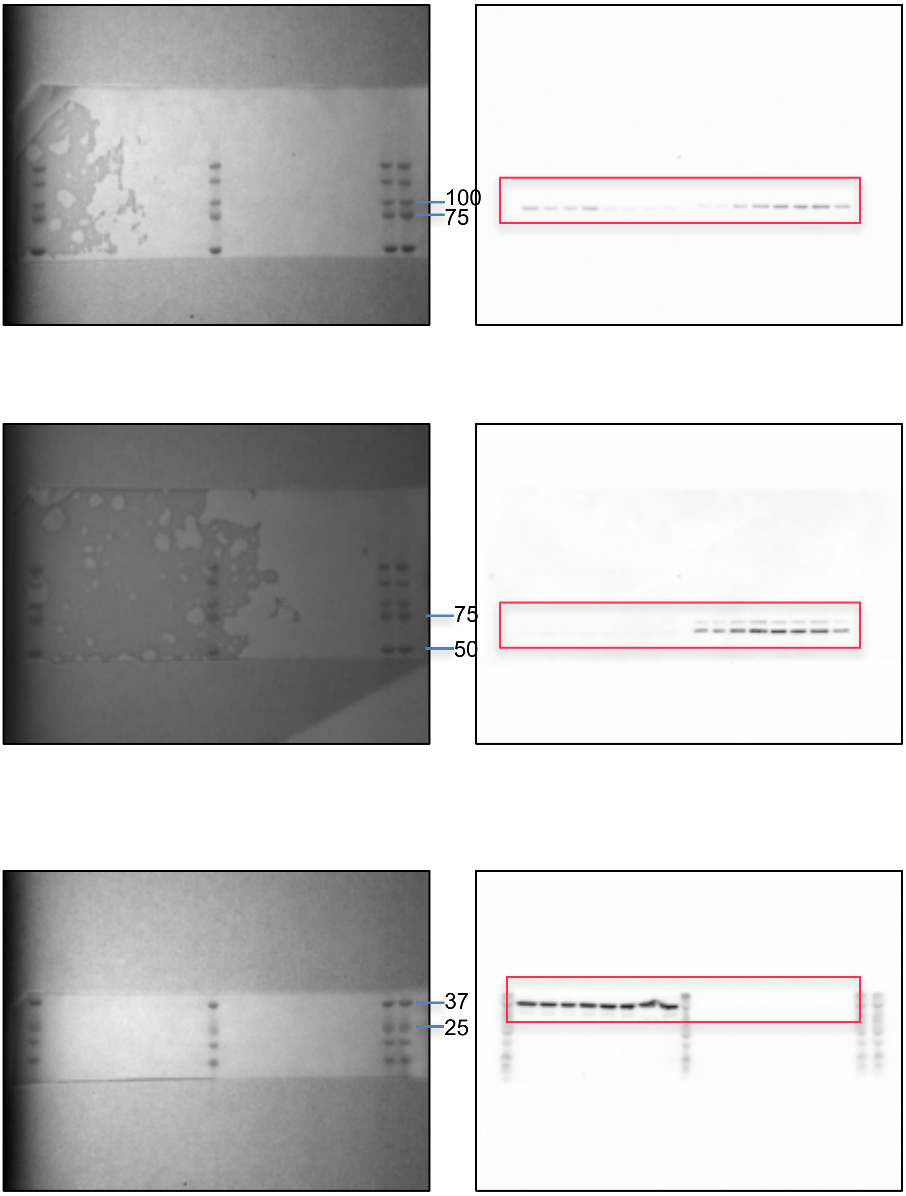


**Supplementary Figure 10. Uncropped blots.** The red sections indicate blot results shown in Figure 2.

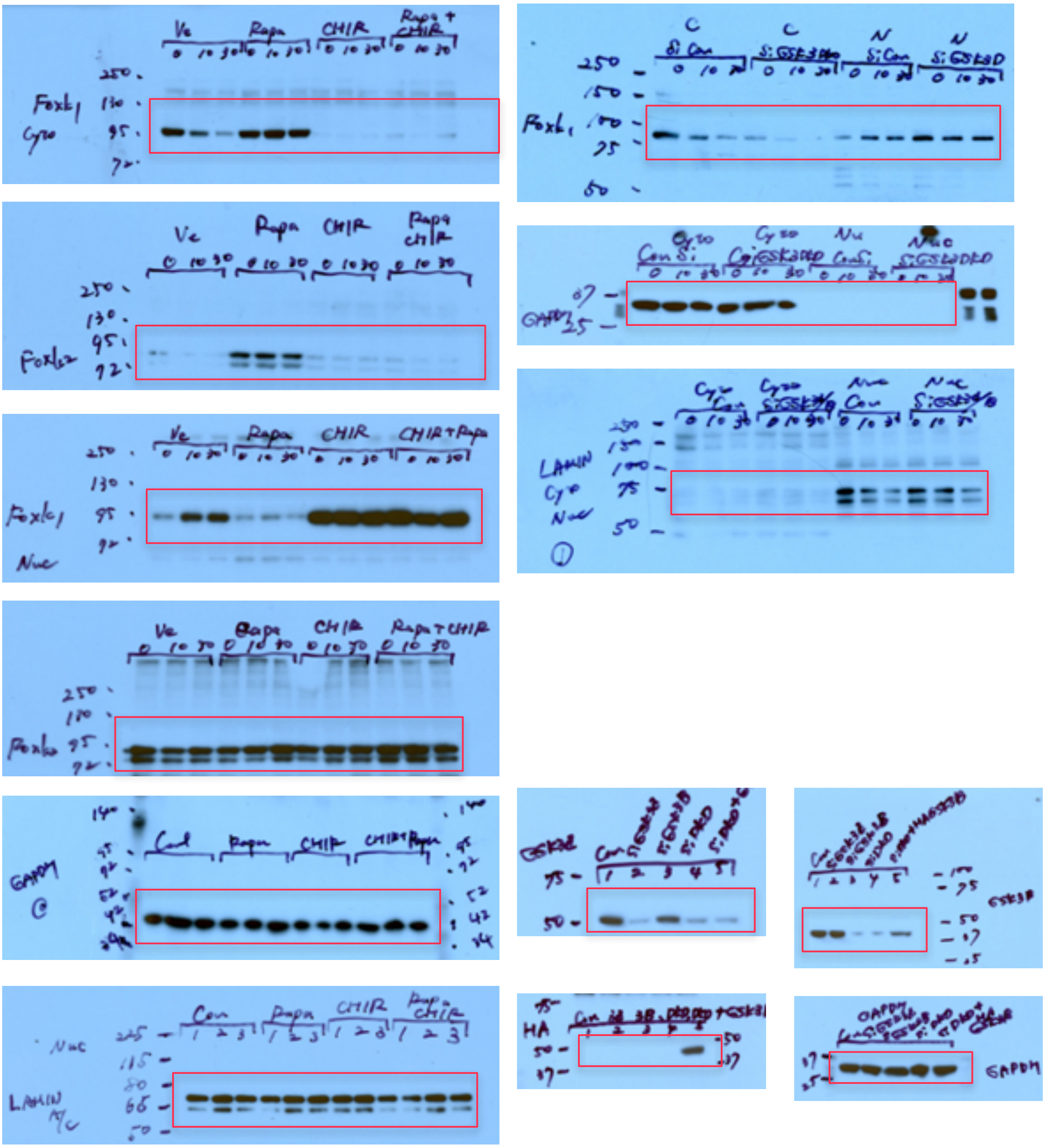

**Supplementary Figure 11. Uncropped blots.** The red sections indicate blot results shown in Figure 3.

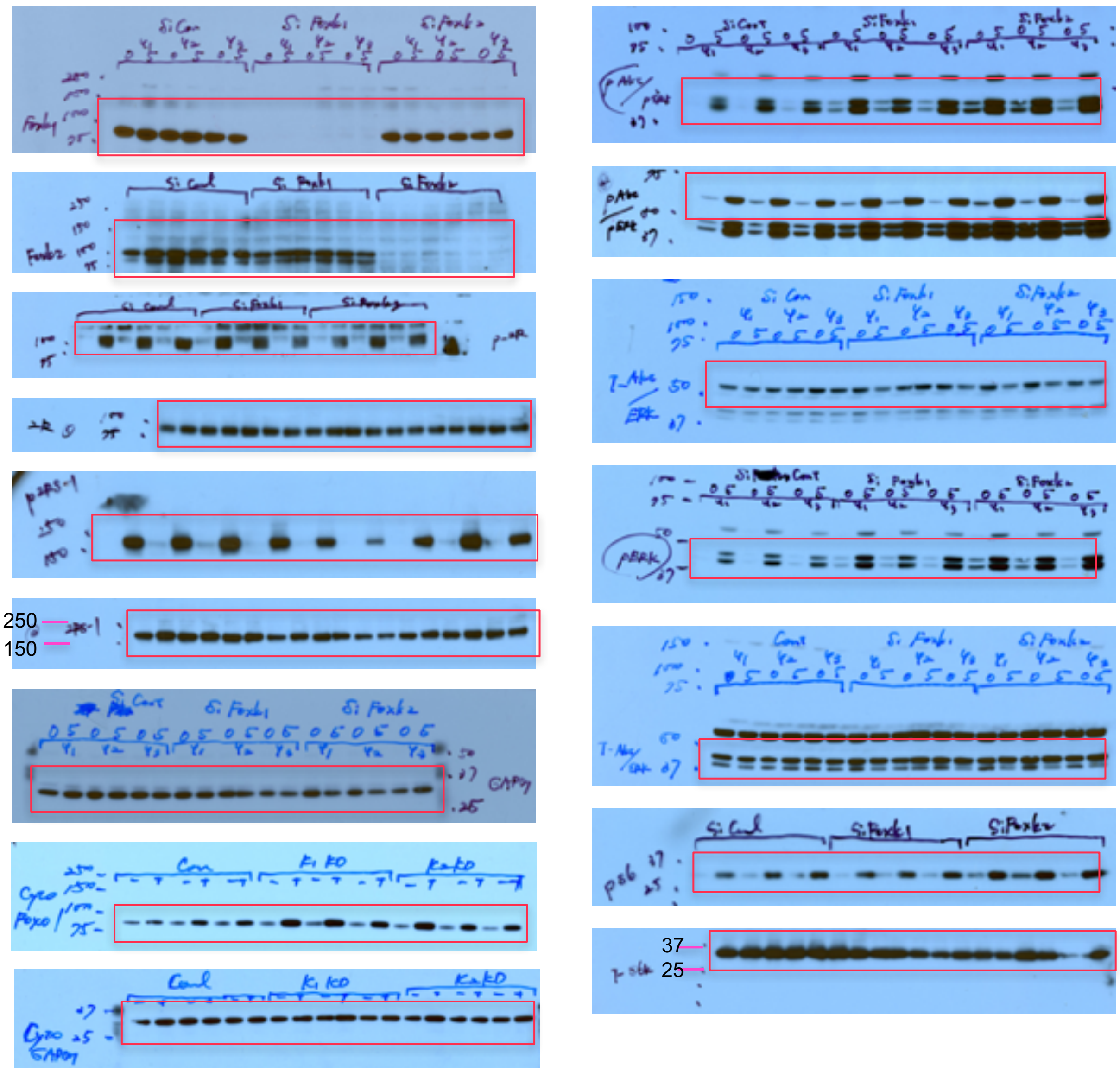


**Supplementary Figure 12. Uncropped blots.** The red sections indicate blot results shown in Figure 4.

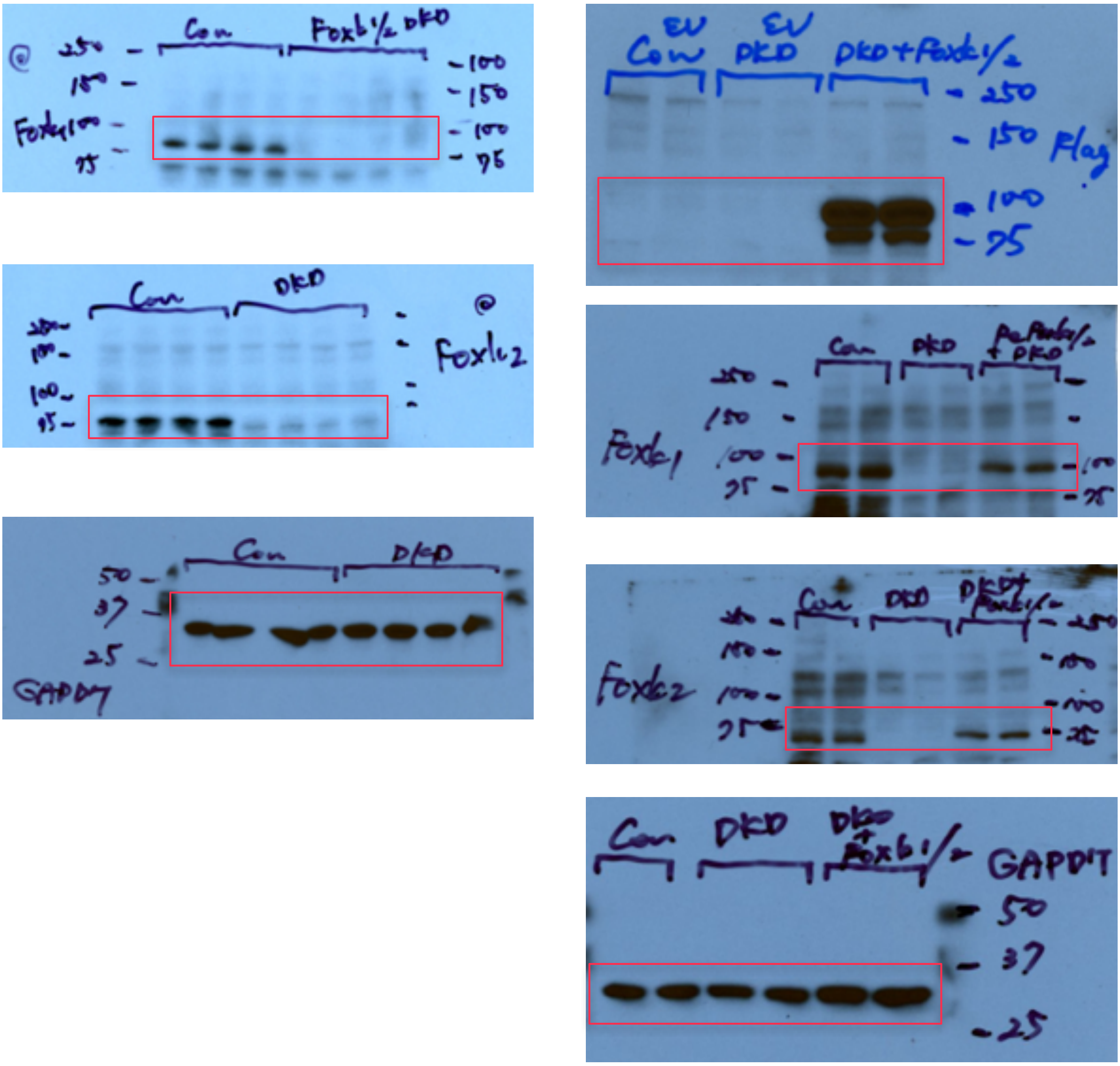


**Supplementary Figure 13. Uncropped blots.** The red sections indicate blot results shown in Figure 5 and 6.

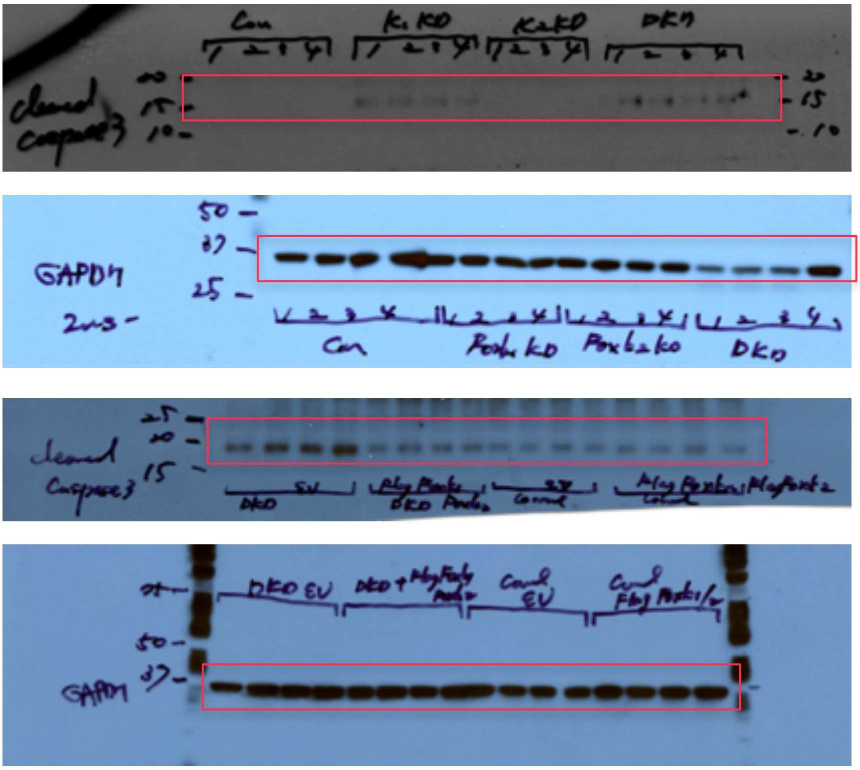


**Supplementary Figure 14. Uncropped blots.** The red sections indicate blot results shown in Figure 7.

**Selected top differentially regulated pathways in Control and DKD cells (Reactome).**

| Pathway | Compared with Control | p-value | FDR |
|---|---|---|---|
| Extrinsic pathway for apoptosis | Up | 6.68.E-09 | 4.33.E-06 |
| Regulation of pyruvate dehydrogenase PDH complex | Down | 1.52.E-06 | 7.00.E-05 |
| Synthesis of very long chain fatty acyl CoAs | Down | 1.52.E-05 | 3.19.E-04 |
| G2 M DNA damage checkpoint | Down | 5.12.E-05 | 8.19.E-04 |
| Apoptotic cleavage of cell adhesion proteins | Down | 1.38.E-04 | 1.82.E-03 |
| Caspase mediated cleavage of cytoskeleton proteins | Up | 2.32.E-04 | 2.60.E-03 |
| Mitotic G2 G2 M phases | Down | 3.06.E-04 | 3.16.E-03 |
| Fatty acid triacyglycerol and ketone body metabolism | Down | 4.10.E-04 | 3.83.E-03 |
| Mitotic M M G1 phases | Down | 8.21.E-04 | 6.34.E-03 |
| Cell cycle | Down | 1.57.E-03 | 9.78.E-03 |
| Cell cycle mitotic | Down | 2.08.E-03 | 1.13.E-02 |
| G2 M checkpoints | Down | 5.45.E-03 | 2.54.E-02 |
| G0 and early G1 | Down | 8.92.E-03 | 3.57.E-02 |
| Triglyceride biosynthesis | Down | 1.10.E-02 | 4.17.E-02 |
| Pyruvate metabolism and citric acid TCA cycle | Down | 1.98.E-02 | 6.26.E-02 |
| Metabolism of lipids and lipoproteins | Down | 2.04.E-02 | 6.39.E-02 |
| Peroxisomal lipid metabolism | Down | 3.20.E-02 | 9.01.E-02 |
| Mitochondrial fatty acid beta oxidation | Down | 3.82.E-02 | 1.04.E-01 |
| Intrinsic pathway for apoptosis | Up | 3.20.E-02 | 8.85.E-02 |
| Apoptosis induced DNA fragmentation | Down | 2.34.E-02 | 6.98.E-02 |

**Sakaguchi et al., Supplementary Table 1**

# Top genes significantly altered in FoxK1KD, FoxK2KD and DKD cells.

| | | | FoxK1KD compared to Control | | | FoxK2KD compared to Control | | | DKD compared to Control | | |
|---|---|---|---|---|---|---|---|---|---|---|---|
| Symbol | Entrez | Name | FC | P-value | FDR | FC | P-value | FDR | FC | P-value | FDR |
| Mcam | 84004 | melanoma cell adhesion molecule | 5.01 | 1.19E-07 | 5.88E-05 | 2.02 | 0.0129 | 0.331 | 3.78 | 7.84E-06 | 0.000244 |
| Lcn2 | 16819 | lipocalin 2 | 1.51 | 0.0128 | 0.163 | 2.72 | 1.77E-08 | 6.10E-05 | 3.50 | 7.36E-11 | 3.18E-08 |
| Fxyd2 | 11936 | FXYD domain-containing ion transport regulator 2 | 2.36 | 1.68E-06 | 0.000527 | 1.93 | 9.34E-05 | 0.0364 | 3.38 | 2.69E-10 | 9.72E-08 |
| Thbs1 | 21825 | thrombospondin 1 | 2.68 | 3.19E-14 | 1.47E-10 | 1.29 | 0.00713 | 0.264 | 2.39 | 1.23E-12 | 1.55E-09 |
| Ptgds | 19215 | prostaglandin D2 synthase (brain) | 1.26 | 0.0275 | 0.239 | 1.40 | 0.00091 | 0.11 | 2.28 | 3.13E-11 | 1.73E-08 |
| Serpina1a | 20700 /// 20703 | serine (or cysteine) peptidase inhibitor, clade A, member 1A | 1.90 | 2.49E-07 | 0.000114 | 1.76 | 1.57E-06 | 0.00309 | 2.20 | 1.57E-09 | 4.25E-07 |
| C3 | 12266 | complement component 3 | 1.27 | 0.0264 | 0.234 | 2.13 | 8.79E-10 | 6.06E-06 | 2.20 | 3.79E-10 | 1.22E-07 |
| 4-Sep | 18952 | septin 4 | 2.13 | 8.56E-13 | 2.95E-09 | 1.41 | 3.51E-05 | 0.0193 | 2.15 | 3.83E-13 | 8.80E-10 |
| Bcam | 57278 | basal cell adhesion molecule | 1.62 | 3.48E-06 | 0.000792 | 1.33 | 0.00201 | 0.155 | 2.13 | 4.96E-11 | 2.44E-08 |
| Saa3 | 20210 | serum amyloid A 3 | 1.63 | 0.00205 | 0.0587 | 2.26 | 5.19E-07 | 0.00119 | 2.07 | 8.23E-06 | 0.000255 |
| Rab11fip5 | 52055 | RAB11 family interacting protein 5 (class I) | 1.62 | 3.86E-07 | 0.000161 | 1.40 | 9.14E-05 | 0.0364 | 2.00 | 3.54E-11 | 1.81E-08 |
| Slc6a18 | 22598 | solute carrier family 6 (neurotransmitter transporter), member 18 | 1.55 | 4.84E-06 | 0.000997 | 1.26 | 0.00664 | 0.259 | 1.99 | 9.21E-11 | 3.85E-08 |
| Serpina1b | 20701 | serine (or cysteine) preptidase inhibitor, clade A, member 1B | 1.63 | 5.67E-06 | 0.00111 | 1.77 | 1.14E-07 | 0.000313 | 1.97 | 3.74E-09 | 7.87E-07 |
| Notch3 | 18131 | notch 3 | 2.07 | 2.35E-09 | 2.95E-06 | 1.21 | 0.0601 | 0.508 | 1.92 | 3.51E-08 | 4.57E-06 |
| Rap1gap | 110351 | Rap1 GTPase-activating protein | 1.72 | 1.92E-06 | 0.000563 | 1.26 | 0.0221 | 0.393 | 1.92 | 3.48E-08 | 4.57E-06 |
| Wdr45 | 54636 | WD repeat domain 45 | 1.40 | 0.000598 | 0.0271 | 1.50 | 2.64E-05 | 0.0182 | 1.90 | 3.77E-09 | 7.87E-07 |
| Bag1 | 12017 | BCL2-associated athanogene 1 | 1.18 | 0.0298 | 0.248 | 1.41 | 1.02E-05 | 0.0128 | 1.87 | 2.30E-11 | 1.47E-08 |
| Gstm1 | 14862 | glutathione S-transferase, mu 1 | 1.31 | 0.00188 | 0.0563 | 1.29 | 0.00243 | 0.168 | 1.85 | 9.55E-10 | 2.69E-07 |
| Stc2 | 20856 | stanniocalcin 2 | 1.39 | 8.07E-05 | 0.00752 | 1.33 | 0.000268 | 0.0637 | 1.81 | 2.75E-10 | 9.72E-08 |
| Myo7a | 17921 | myosin VIIA | 1.68 | 8.54E-07 | 0.00031 | 1.44 | 0.000149 | 0.0438 | 1.75 | 1.66E-07 | 1.46E-05 |
| Alpl | 11647 | alkaline phosphatase, liver/bone/kidney | 1.70 | 9.15E-07 | 0.000324 | 1.31 | 0.00436 | 0.219 | 1.67 | 1.35E-06 | 6.47E-05 |
| Lgals3bp | 19039 | lectin, galactoside-binding, soluble, 3 binding protein | 1.13 | 0.0269 | 0.236 | 1.28 | 9.16E-06 | 0.0126 | 1.65 | 1.08E-12 | 1.55E-09 |
| Ccnd1 | 12443 | cyclin D1 | 1.55 | 1.93E-11 | 4.43E-08 | 1.24 | 3.42E-05 | 0.0193 | 1.59 | 1.22E-12 | 1.55E-09 |
| Adgrg1 | 14766 | adhesion G protein-coupled receptor G1 | 1.52 | 7.29E-09 | 6.70E-06 | 1.12 | 0.0484 | 0.486 | 1.56 | 8.33E-10 | 2.42E-07 |
| Fhod3 | 225288 | formin homology 2 domain containing 3 | 1.79 | 1.12E-11 | 3.09E-08 | 1.23 | 0.00179 | 0.146 | 1.55 | 1.60E-08 | 2.40E-06 |
| Dab2 | 13132 | disabled 2, mitogen-responsive phosphoprotein | 2.00 | 6.53E-11 | 1.13E-07 | 1.15 | 0.0969 | 0.571 | 1.54 | 3.69E-06 | 0.000142 |
| Sgk1 | 20393 | serum/glucocorticoid regulated kinase 1 | 1.47 | 2.20E-06 | 0.000633 | 1.26 | 0.00136 | 0.13 | 1.46 | 2.47E-06 | 0.000104 |
| Lbp | 16803 | lipopolysaccharide binding protein | 1.36 | 3.37E-07 | 0.00015 | 1.10 | 0.0558 | 0.503 | 1.41 | 9.82E-09 | 1.76E-06 |
| Lss | 16987 | lanosterol synthase | 0.74 | 6.29E-08 | 4.27E-05 | 0.88 | 0.00618 | 0.256 | 0.81 | 2.61E-05 | 0.000585 |
| Tgoln1 | 22134 /// 22135 | trans-golgi network protein | 0.69 | 3.12E-09 | 3.58E-06 | -0.92 | 0.0656 | 0.518 | 0.76 | 1.52E-06 | 7.15E-05 |
| Acat2 | 110460 | acetyl-Coenzyme A acetyltransferase 2 | 0.65 | 4.78E-09 | 5.07E-06 | 0.88 | 0.0262 | 0.411 | 0.75 | 6.92E-06 | 0.000223 |
| Abcc2 | 12780 | ATP-binding cassette, sub-family C (CFTR/MRP), member 2 | 0.81 | 5.65E-05 | 0.00604 | 0.87 | 0.00197 | 0.153 | 0.69 | 3.42E-10 | 1.12E-07 |
| Gldc | 104174 | glycine decarboxylase | 0.79 | 0.000109 | 0.00906 | 0.82 | 0.000486 | 0.0918 | 0.69 | 4.02E-08 | 5.03E-06 |
| Ssr3 | 67437 | signal sequence receptor, gamma | 0.79 | 0.000421 | 0.0216 | 0.83 | 0.00141 | 0.134 | 0.66 | 1.34E-08 | 2.12E-06 |
| Tspan12 | 269831 | tetraspanin 12 | 0.82 | 0.000623 | 0.0278 | 0.83 | 0.00119 | 0.127 | 0.65 | 2.97E-10 | 1.03E-07 |
| Scd2 | 20250 | stearoyl-Coenzyme A desaturase 2 | 0.85 | 0.000528 | 0.025 | 0.87 | 0.000911 | 0.11 | 0.64 | 5.65E-14 | 1.56E-10 |
| Chmp2b | 68942 | charged multivesicular body protein 2B | 0.82 | 0.000891 | 0.0347 | 0.86 | 0.00836 | 0.284 | 0.64 | 4.29E-10 | 1.32E-07 |
| Top2a | 21973 | topoisomerase (DNA) II alpha | 0.79 | 6.65E-05 | 0.00674 | 0.79 | 4.69E-05 | 0.0239 | 0.63 | 5.28E-11 | 2.51E-08 |
| Tfrc | 22042 | transferrin receptor | 0.80 | 9.12E-05 | 0.00811 | 0.85 | 0.00193 | 0.152 | 0.62 | 1.20E-11 | 9.22E-09 |
| Foxk1 | 17425 | forkhead box K1 | 0.48 | 3.96E-11 | 7.80E-08 | 0.87 | 0.0531 | 0.498 | 0.59 | 3.27E-08 | 4.37E-06 |
| Cnih1 | 12793 | cornichon family AMPA receptor auxiliary protein 1 | 0.82 | 0.00267 | 0.0709 | 0.85 | 0.00776 | 0.273 | 0.59 | 5.83E-11 | 2.68E-08 |
| Aff4 | 93736 | AF4/FMR2 family, member 4 | 0.70 | 5.39E-09 | 5.31E-06 | 0.91 | 0.0514 | 0.493 | 0.58 | 3.75E-14 | 1.56E-10 |
| Scd1 | 20249 | stearoyl-Coenzyme A desaturase 1 | 0.56 | 2.08E-14 | 1.43E-10 | 0.85 | 0.00176 | 0.146 | 0.57 | 5.24E-14 | 1.56E-10 |
| Vcam1 | 22329 | vascular cell adhesion molecule 1 | 0.51 | 5.57E-16 | 7.68E-12 | -0.93 | 0.186 | 0.681 | 0.56 | 1.75E-14 | 1.21E-10 |
| Ube2d3 | 66105 | ubiquitin-conjugating enzyme E2D 3 | 0.89 | 0.078 | 0.387 | 0.75 | 1.85E-05 | 0.0182 | 0.54 | 2.36E-12 | 2.51E-09 |
| Cmtm8 | 70031 | CKLF-like MARVEL transmembrane domain containing 8 | 0.67 | 5.61E-05 | 0.00604 | 0.78 | 0.00448 | 0.219 | 0.51 | 8.35E-09 | 1.56E-06 |
| Hmgn5 | 50887 | high-mobility group nucleosome binding domain 5 | 0.63 | 0.000394 | 0.0209 | 0.56 | 8.42E-06 | 0.0126 | 0.48 | 6.62E-07 | 3.95E-05 |
| mt-Nd6 | 17722 | mitochondrially encoded NADH dehydrogenase 6 | 0.90 | 0.158 | 0.52 | 0.74 | 9.45E-05 | 0.0364 | 0.47 | 1.23E-12 | 1.55E-09 |
| Sprr1a | 20753 | small proline-rich protein 1A | 0.35 | 6.86E-08 | 4.27E-05 | 0.59 | 0.000203 | 0.0509 | 0.46 | 4.18E-06 | 0.000153 |
| Foxk2 | 68837 | forkhead box K2 | 0.84 | 0.00574 | 0.108 | 0.34 | 2.26E-20 | 3.12E-16 | 0.36 | 4.80E-19 | 6.61E-15 |

**Sakaguchi et al., Supplementary Table 2**

Selected common regulated pathways by insulin stimulation in Control and DKD cells (Reactome).

| | Control insulin + vs - | | | DKD insulin + vs - | | | DKD vs Control | |
|---|---|---|---|---|---|---|---|---|
| | | p-value | FDR | | p-value | FDR | p-value | FDR |
| Glucose transport | Up | 6.37E-13 | 4.14E-10 | Up | 6.18E-13 | 2.01E-10 | 0.301 | 0.785 |
| Cholesterol biosynthesis | Up | 1.12E-10 | 1.82E-08 | Up | 2.19E-12 | 2.8E-10 | 0.508 | 0.86 |
| Metabolism of carbohydrates | Up | 2.93E-10 | 0.000000025 | Up | 2.19E-10 | 1.78E-08 | 0.402 | 0.807 |
| Diabetes pathways | Up | 3.08E-10 | 0.000000025 | Up | 1.33E-10 | 1.23E-08 | 0.601 | 0.9 |
| Triglyceride biosynthesis | Up | 0.00000232 | 0.0000334 | Up | 3.83E-09 | 0.000000118 | 0.169 | 0.636 |
| Glycolysis | Up | 0.0000132 | 0.00014 | Up | 0.000000802 | 0.0000102 | 0.724 | 0.956 |
| Glucose metabolism | Up | 0.0000742 | 0.000618 | Up | 0.000000278 | 0.00000475 | 0.262 | 0.761 |
| Metabolism of vitamins and cofactors | Up | 0.00015 | 0.00114 | Up | 1.03E-08 | 0.000000277 | 0.0174 | 0.303 |
| Integration of energy metabolism | Up | 0.000179 | 0.0013 | Up | 0.0000085 | 0.0000788 | 0.625 | 0.911 |
| Metabolism of amino acids and derivatives | Up | 0.000461 | 0.00285 | Up | 0.00103 | 0.00455 | 0.667 | 0.933 |

**Sakaguchi et al., Supplementary Table 3**

Top significant genes that were differentially regulated in Control, FoxK1KD, FoxK2KD and DKD cells in response to insulin stimulation.

| Symbol | Entrez | Name | Fold change to basal | | | | P-value (Compared to Control) | | |
|---|---|---|---|---|---|---|---|---|---|
| | | | Control | FoxK1KD | FoxK2KD | DKD | FoxK1KD | FoxK2KD | DKD |
| Gpnmb | 93695 | glycoprotein (transmembrane) nmb | 22.20 | 7.66 | 15.40 | 2.11 | 0.207 | 0.638 | 0.000959 |
| Gm5160 | | predicted gene 5160 | 8.96 | 5.26 | 5.01 | 0.91 | 0.434 | 0.361 | 0.000536 |
| Aqp9 | 64008 | aquaporin 9 | 6.72 | 1.65 | 1.38 | 1.24 | 0.0595 | 0.024 | 0.0258 |
| Ppp1ca | 19045 | protein phosphatase 1, catalytic subunit, alpha isoform | 6.22 | 5.67 | 6.74 | 1.05 | 0.884 | 0.896 | 0.00398 |
| Aldoart1 | 353204 | aldolase 1 A, retrogene 1 | 6.03 | 2.97 | 2.47 | 0.69 | 0.322 | 0.138 | 0.00044 |
| Spry4 | 24066 | sprouty homolog 4 (Drosophila) | 5.72 | 2.43 | 2.36 | 1.29 | 0.112 | 0.092 | 0.00765 |
| Sstr2 | 20606 | somatostatin receptor 2 | 4.86 | 0.90 | 0.86 | 0.82 | 0.035 | 0.0228 | 0.0267 |
| Ddc | 13195 | dopa decarboxylase | 4.85 | 1.63 | 0.79 | 0.56 | 0.187 | 0.0198 | 0.00877 |
| Ptpn5 | 19259 | protein tyrosine phosphatase, non-receptor type 5 | 4.75 | 1.43 | 2.67 | 0.69 | 0.15 | 0.459 | 0.0272 |
| Gm8797 | | predicted pseudogene 8797 | 4.35 | 5.08 | 1.47 | 0.57 | 0.841 | 0.152 | 0.00799 |
| Ass1 | 11898 | argininosuccinate synthetase 1 | 4.31 | 0.99 | 0.48 | 0.69 | 0.0712 | 0.00826 | 0.0321 |
| Gcnt3 | 72077 | glucosaminyl (N-acetyl) transferase 3, mucin type | 4.29 | 1.38 | 1.55 | 1.35 | 0.0274 | 0.0728 | 0.0323 |
| Gm11808 | 22186 | predicted gene 11808 | 4.19 | 5.28 | 2.08 | 0.52 | 0.726 | 0.271 | 0.00154 |
| Gstm6 | 14867 | glutathione S-transferase, mu 6 | 4.08 | 0.86 | 0.99 | 0.81 | 0.0255 | 0.0249 | 0.0195 |
| Vtn | 22370 | vitronectin | 3.75 | 1.35 | 0.72 | 0.68 | 0.215 | 0.0273 | 0.0334 |
| Ftl1-ps1 | | ferritin light polypeptide 1, pseudogene 1 | 3.41 | 3.65 | 2.80 | 1.18 | 0.789 | 0.403 | 0.0000357 |
| Chrd | 12667 | chordin | 3.38 | 1.26 | 1.60 | 0.78 | 0.0967 | 0.2 | 0.0217 |
| Arntl2 | 272322 | aryl hydrocarbon receptor nuclear translocator-like 2 | 3.30 | 1.92 | 1.77 | 0.54 | 0.473 | 0.469 | 0.0328 |
| Zfp775 | 243372 | zinc finger protein 775 | 3.24 | 0.42 | 1.32 | 0.53 | 0.00476 | 0.163 | 0.00746 |
| Fam222a | 433940 | family with sequence similarity 222, member A | 3.18 | 0.93 | 1.37 | 0.88 | 0.0194 | 0.0895 | 0.0151 |
| Ccne1 | 12447 | cyclin E1 | 3.17 | 3.44 | 2.41 | 1.85 | 0.713 | 0.187 | 0.017 |
| Gale | 74246 | galactose-4-epimerase, UDP | 2.96 | 3.28 | 3.02 | 1.80 | 0.612 | 0.91 | 0.0128 |
| Ccr9 | 12769 | chemokine (C-C motif) receptor 9 | 2.95 | 1.56 | 2.09 | 0.36 | 0.319 | 0.571 | 0.00479 |
| Nutf2 | 68051 /// 621832 | nuclear transport factor 2 | 2.85 | 1.27 | 1.77 | 0.93 | 0.0968 | 0.308 | 0.0209 |
| Dusp5 | 240672 | dual specificity phosphatase 5 | 2.80 | 1.50 | 1.12 | 0.74 | 0.0961 | 0.0114 | 0.00113 |
| Pnldc1 | 240023 | poly(A)-specific ribonuclease (PARN)-like domain containing 1 | 0.33 | 1.88 | 1.64 | 1.77 | 0.0234 | 0.0457 | 0.0365 |
| Cyp2d26 | 76279 | cytochrome P450, family 2, subfamily d, polypeptide 26 | 0.32 | 2.48 | 2.80 | 1.23 | 0.0223 | 0.0122 | 0.106 |
| Nupr1l | 69034 | nuclear protein transcriptional regulator 1 like | 0.31 | 0.56 | 0.51 | 1.19 | 0.431 | 0.502 | 0.0833 |
| Ikzf4 | 22781 | IKAROS family zinc finger 4 | 0.30 | 1.41 | 0.32 | 1.24 | 0.0805 | 0.929 | 0.0932 |
| Nat14 | 269854 | N-acetyltransferase 14 | 0.30 | 0.76 | 0.65 | 0.74 | 0.0496 | 0.0882 | 0.0525 |
| Fhl1 | 14199 | four and a half LIM domains 1 | 0.30 | 0.78 | 0.81 | 1.15 | 0.114 | 0.0791 | 0.0228 |
| Gprin3 | 243385 | GPRIN family member 3 | 0.29 | 1.18 | 1.51 | 1.38 | 0.076 | 0.0353 | 0.0319 |
| Fn3k | 63828 | fructosamine 3 kinase | 0.29 | 0.47 | 0.41 | 0.53 | 0.0853 | 0.22 | 0.0382 |
| Hist1h4i | 319158 | histone cluster 1, H4i | 0.28 | 1.17 | 1.40 | 1.31 | 0.00979 | 0.00501 | 0.00615 |
| Fbxo32 | 67731 | F-box protein 32 | 0.28 | 0.31 | 0.26 | 0.38 | 0.36 | 0.606 | 0.00631 |
| Zfp960 | 449000 | zinc finger protein 960 | 0.27 | 1.78 | 1.17 | 2.12 | 0.0273 | 0.0582 | 0.0112 |
| Ttc6 | 70846 | tetratricopeptide repeat domain 6 | 0.26 | 1.00 | 0.28 | 1.23 | 0.0324 | 0.911 | 0.026 |
| Tcf7l1 | 21415 | transcription factor 7 like 1 (T cell specific, HMG box) | 0.26 | 1.32 | 0.71 | 0.85 | 0.021 | 0.139 | 0.0939 |
| Slc52a3 | 69698 | solute carrier protein family 52, member 3 | 0.25 | 0.96 | 1.06 | 1.74 | 0.0875 | 0.0531 | 0.0118 |
| Mturn | 68235 | maturin, neural progenitor differentiation regulator homolog (Xenopus) | 0.24 | 0.34 | 0.30 | 0.43 | 0.0593 | 0.185 | 0.00215 |
| Dclk3 | 245038 | doublecortin-like kinase 3 | 0.24 | 0.40 | 0.40 | 0.42 | 0.148 | 0.156 | 0.105 |
| Gpr160 | 71862 | G protein-coupled receptor 160 | 0.23 | 0.75 | 0.53 | 0.99 | 0.0394 | 0.136 | 0.0128 |
| Xkrx | 331524 | X-linked Kx blood group related, X-linked | 0.22 | 0.72 | 0.75 | 2.25 | 0.14 | 0.129 | 0.00712 |
| Me3 | 109264 | malic enzyme 3, NADP(+)-dependent, mitochondrial | 0.22 | 0.52 | 0.47 | 0.56 | 0.0333 | 0.0646 | 0.0237 |
| Chst4 | 26887 | carbohydrate (chondroitin 6/keratan) sulfotransferase 4 | 0.20 | 1.16 | 0.81 | 0.84 | 0.0362 | 0.0927 | 0.0859 |
| Hoxb6 | 15414 | homeobox B6 | 0.19 | 1.01 | 1.39 | 1.68 | 0.0169 | 0.00455 | 0.00596 |
| H2-T23 | 15040 | histocompatibility 2, T region locus 23 | 0.17 | 0.50 | 0.68 | 0.53 | 0.115 | 0.0379 | 0.0993 |
| Slamf9 | 98365 | SLAM family member 9 | 0.15 | 0.91 | 0.57 | 0.93 | 0.0233 | 0.0951 | 0.0241 |
| Corin | 53419 | corin | 0.14 | 0.35 | 0.46 | 0.44 | 0.128 | 0.0468 | 0.0543 |
| C330021F23Rik | 546049 | RIKEN cDNA C330021F23 gene | 0.12 | 0.52 | 1.12 | 0.59 | 0.0318 | 0.00197 | 0.0226 |

**Sakaguchi et al., Supplementary Table 4**

# Comparison of mitochondrial related genes in Control, FoxK1KD, FoxK2KD and DKD cells.

| Symbol | Entrez | Name | FoxK1KD compared to Control FC | FoxK1KD compared to Control P-value | FoxK2KD compared to Control FC | FoxK2KD compared to Control P-value | DKD compared to Control FC | DKD compared to Control P-value |
|---|---|---|---|---|---|---|---|---|
| Bckdha | 12039 | branched chain ketoacid dehydrogenase E1, alpha polypeptide | 1.17 | 0.363 | 1.19 | 0.297 | 2.10 | 0.0000244 |
| Rab11fip5 | 52055 | RAB11 family interacting protein 5 (class I) | 1.62 | 0.000000386 | 1.40 | 0.0000914 | 2.00 | 3.54E-11 |
| Mtch1 | 56462 | mitochondrial carrier 1 | 0.88 | 0.0735 | 1.09 | 0.178 | 1.51 | 0.000000102 |
| Ndufs8 | 225887 | NADH dehydrogenase (ubiquinone) Fe-S protein 8 | 1.11 | 0.24 | 1.22 | 0.0188 | 1.50 | 0.0000147 |
| Pink1 | 68943 | PTEN induced putative kinase 1 | 1.04 | 0.641 | 1.17 | 0.0744 | 1.48 | 0.0000354 |
| Bbc3 | 170770 | BCL2 binding component 3 | 1.08 | 0.322 | 1.06 | 0.476 | 1.41 | 0.0000582 |
| Casp7 | 12369 | caspase 7 | 0.75 | 0.00212 | 1.04 | 0.627 | 1.41 | 0.000115 |
| Phb | 18673 /// 237880 | prohibitin | 0.93 | 0.371 | 1.11 | 0.221 | 1.39 | 0.000349 |
| Pgs1 | 74451 | phosphatidylglycerophosphate synthase 1 | 1.19 | 0.0217 | 1.15 | 0.0489 | 1.38 | 0.0000295 |
| Mfn2 | 170731 | mitofusin 2 | 1.20 | 0.00605 | 1.08 | 0.201 | 1.37 | 0.0000062 |
| Me3 | 109264 | malic enzyme 3, NADP(+)-dependent, mitochondrial | 1.44 | 0.0301 | 0.95 | 0.786 | 1.35 | 0.0731 |
| Fis1 | 66437 | fission, mitochondrial 1 | 0.94 | 0.397 | 1.11 | 0.123 | 1.31 | 0.000359 |
| Slc25a22 | 68267 | solute carrier family 25 (mitochondrial carrier, glutamate), member 22 | 1.06 | 0.48 | 1.01 | 0.894 | 1.31 | 0.00117 |
| Letm1 | 56384 | leucine zipper-EF-hand containing transmembrane protein 1 | 0.96 | 0.51 | 0.92 | 0.167 | 1.25 | 0.000402 |
| Phb2 | 12034 | prohibitin 2 | 0.84 | 0.033 | 1.19 | 0.0202 | 1.24 | 0.00523 |
| Shmt2 | 108037 | serine hydroxymethyltransferase 2 (mitochondrial) | 0.74 | 0.000569 | 0.92 | 0.24 | 1.22 | 0.0114 |
| Idh2 | 269951 | isocitrate dehydrogenase 2 (NADP+), mitochondrial | 1.04 | 0.529 | 1.13 | 0.03 | 1.21 | 0.000768 |
| Tbrg4 | 21379 | transforming growth factor beta regulated gene 4 | 0.89 | 0.102 | 1.03 | 0.655 | 1.21 | 0.00426 |
| Bphl | 68021 | biphenyl hydrolase-like (serine hydrolase, breast epithelial mucin-associated antigen) | 1.20 | 0.0213 | 1.11 | 0.173 | 1.19 | 0.0223 |
| Glyat | 107146 | glycine-N-acyltransferase | 1.48 | 0.0999 | 1.27 | 0.296 | 1.12 | 0.658 |
| Cox18 | 231430 | cytochrome c oxidase assembly protein 18 | 0.74 | 0.0744 | 1.09 | 0.546 | 1.11 | 0.481 |
| Timm50 | 66525 | translocase of inner mitochondrial membrane 50 | 0.77 | 0.000841 | 1.04 | 0.596 | 1.11 | 0.116 |
| Abcf2 | 27407 | ATP-binding cassette, sub-family F (GCN20), member 2 | 0.90 | 0.0635 | 1.11 | 0.0509 | 1.10 | 0.0762 |
| Msrb3 | 320183 | methionine sulfoxide reductase B3 | 1.16 | 0.0783 | 1.00 | 0.974 | 1.10 | 0.25 |
| Slc25a11 | 67863 | solute carrier family 25 (mitochondrial carrier oxoglutarate carrier), member 11 | 0.94 | 0.375 | 1.05 | 0.448 | 1.07 | 0.295 |
| Mthfd1 | 108156 | methylenetetrahydrofolate dehydrogenase (NADP+ dependent), methenyltetrahydrofolate cyclohydrolase, formyltetrahydrofolate synthase | 0.96 | 0.455 | 0.99 | 0.867 | 1.04 | 0.444 |
| Ogdh | 18293 | oxoglutarate (alpha-ketoglutarate) dehydrogenase (lipoamide) | 0.98 | 0.673 | 0.92 | 0.0861 | 0.94 | 0.277 |
| Cs | 12974 | citrate synthase | 0.94 | 0.22 | 0.94 | 0.233 | 0.91 | 0.0565 |
| Amacr | 17117 | alpha-methylacyl-CoA racemase | 0.78 | 0.0018 | 0.85 | 0.0216 | 0.91 | 0.206 |
| Hscb | 100900 | HscB iron-sulfur cluster co-chaperone | 1.03 | 0.782 | 1.09 | 0.358 | 0.90 | 0.311 |
| Acadsb | 66885 | acyl-Coenzyme A dehydrogenase, short/branched chain | 1.15 | 0.0304 | 1.05 | 0.41 | 0.88 | 0.0352 |
| Ptrf | 19285 | polymerase I and transcript release factor | 0.87 | 0.0562 | 0.83 | 0.00887 | 0.88 | 0.058 |
| Supv3l1 | 338359 | suppressor of var1, 3-like 1 (S. cerevisiae) | 0.94 | 0.42 | 0.97 | 0.727 | 0.86 | 0.0474 |
| Gpx4 | 625249 | glutathione peroxidase 4 | 0.94 | 0.462 | 1.25 | 0.00525 | 0.85 | 0.0519 |
| Acadm | 11364 | acyl-Coenzyme A dehydrogenase, medium chain | 1.01 | 0.869 | 1.01 | 0.846 | 0.84 | 0.00406 |
| Vdac1 | 22333 | voltage-dependent anion channel 1 | 0.99 | 0.831 | 0.97 | 0.531 | 0.80 | 0.0000363 |
| Slc25a13 | 50799 | solute carrier family 25 (mitochondrial carrier, adenine nucleotide translocator), member 13 | 0.85 | 0.00154 | 1.03 | 0.589 | 0.79 | 0.0000118 |
| Pdha1 | 18597 | pyruvate dehydrogenase E1 alpha 1 | 0.93 | 0.263 | 1.00 | 0.937 | 0.78 | 0.000271 |
| Oxsm | 71147 | 3-oxoacyl-ACP synthase, mitochondrial | 0.92 | 0.267 | 0.93 | 0.27 | 0.78 | 0.00156 |
| Mut | 17850 | methylmalonyl-Coenzyme A mutase | 0.96 | 0.527 | 0.93 | 0.184 | 0.77 | 0.0000381 |
| Abce1 | 24015 | ATP-binding cassette, sub-family E (OABP), member 1 | 0.89 | 0.0623 | 0.86 | 0.0132 | 0.76 | 0.0000329 |
| Clpx | 270166 | caseinolytic mitochondrial matrix peptidase chaperone subunit | 0.88 | 0.0554 | 1.02 | 0.769 | 0.75 | 0.0000336 |
| Asah2 | 54447 | N-acylsphingosine amidohydrolase 2 | 1.12 | 0.211 | 1.04 | 0.603 | 0.75 | 0.00288 |
| Pdk1 | 228026 | pyruvate dehydrogenase kinase, isoenzyme 1 | 0.76 | 0.0000736 | 0.90 | 0.0754 | 0.67 | 0.000000117 |
| Dld | 13382 | dihydrolipoamide dehydrogenase | 0.89 | 0.0857 | 0.90 | 0.0781 | 0.67 | 4.65E-08 |
| Tomm22 | 223696 | translocase of outer mitochondrial membrane 22 homolog (yeast) | 0.79 | 0.00579 | 0.87 | 0.0676 | 0.67 | 0.00000429 |
| Mthfd2 | 17768 | methylenetetrahydrofolate dehydrogenase (NAD+ dependent), methenyltetrahydrofolate cyclohydrolase | 0.70 | 0.000517 | 0.86 | 0.0942 | 0.65 | 0.0000653 |
| Fpgs | 14287 | folylpolyglutamyl synthetase | 0.78 | 0.00681 | 0.92 | 0.289 | 0.65 | 0.0000176 |
| Coq4 | 227683 | coenzyme Q4 | 0.74 | 0.00611 | 0.72 | 0.00231 | 0.63 | 0.000104 |
| Bnip3 | 12176 | BCL2/adenovirus E1B interacting protein 3 | 0.73 | 0.00385 | 0.89 | 0.24 | 0.53 | 0.000000583 |

**Sakaguchi et al., Supplementary Table 5**

# Changes of genes associated with mitochondrial oxidative phosphorylation in Control, FoxK1KD, FoxK2KD and DKD cells.

| Symbol | Entrez | Name | FoxK1KD compared to Control | | FoxK2KD compared to Control | | DKD compared to Control | |
|---|---|---|---|---|---|---|---|---|
| | | | FC | P-value | FC | P-value | FC | P-value |
| Ndufa11 | 69875 /// 239760 | NADH dehydrogenase (ubiquinone) 1 alpha subcomplex 11 | 1.89 | 0.0147 | 2.04 | 0.0043 | 2.24 | 0.0019 |
| Ndufb7 | 66916 | NADH dehydrogenase (ubiquinone) 1 beta subcomplex, 7 | 0.81 | 0.21 | 0.99 | 0.932 | 2.01 | 1.73E-05 |
| Atp6v0a4 | 140494 | ATPase, H+ transporting, lysosomal V0 subunit A4 | 1.55 | 0.00454 | 1.10 | 0.517 | 1.64 | 0.00146 |
| Ndufs8 | 225887 | NADH dehydrogenase (ubiquinone) Fe-S protein 8 | 1.11 | 0.24 | 1.22 | 0.0188 | 1.50 | 1.47E-05 |
| Atp5g2 | 67942 | ATP synthase, H+ transporting, mitochondrial F0 complex, subunit C2 (subunit 9) | 0.79 | 0.023 | 0.85 | 0.0873 | 1.35 | 0.00199 |
| Tcirg1 | 27060 | T cell, immune regulator 1, ATPase, H+ transporting, lysosomal V0 protein A3 | 1.17 | 0.0168 | 1.01 | 0.892 | 1.34 | 1.79E-05 |
| Sdhc | 66052 | succinate dehydrogenase complex, subunit C, integral membrane protein | 0.97 | 0.743 | 1.12 | 0.231 | 1.33 | 0.00435 |
| Cox6b1 | 110323 | cytochrome c oxidase, subunit VIb polypeptide 1 | 0.72 | 0.0368 | 1.08 | 0.591 | 1.32 | 0.0491 |
| Atp6v0b | 114143 | ATPase, H+ transporting, lysosomal V0 subunit B | 0.92 | 0.225 | 0.99 | 0.915 | 1.30 | 0.000401 |
| Ndufv1 | 17995 | NADH dehydrogenase (ubiquinone) flavoprotein 1 | 0.89 | 0.101 | 0.95 | 0.473 | 1.24 | 0.0015 |
| Sdhb | 67680 | succinate dehydrogenase complex, subunit B, iron sulfur (Ip) | 0.92 | 0.429 | 1.17 | 0.124 | 1.21 | 0.0726 |
| Atp6v1f | 66144 | ATPase, H+ transporting, lysosomal V1 subunit F | 0.97 | 0.736 | 1.35 | 0.00189 | 1.20 | 0.0595 |
| Cox5b | 12859 | cytochrome c oxidase subunit Vb | 1.07 | 0.506 | 1.35 | 0.00284 | 1.18 | 0.116 |
| Atp6v1b2 | 11966 | ATPase, H+ transporting, lysosomal V1 subunit B2 | 0.97 | 0.464 | 1.07 | 0.117 | 1.15 | 0.00267 |
| Sdha | 66945 | succinate dehydrogenase complex, subunit A, flavoprotein (Fp) | 0.93 | 0.114 | 0.97 | 0.573 | 1.14 | 0.00875 |
| Ndufc2 | 68197 | NADH dehydrogenase (ubiquinone) 1, subcomplex unknown, 2 | 0.80 | 0.117 | 1.13 | 0.327 | 1.14 | 0.323 |
| Atp6v1g1 | 66290 | ATPase, H+ transporting, lysosomal V1 subunit G1 | 0.97 | 0.657 | 1.02 | 0.77 | 1.12 | 0.108 |
| Ndufb9 | 66218 | NADH dehydrogenase (ubiquinone) 1 beta subcomplex, 9 | 0.87 | 0.119 | 1.09 | 0.283 | 1.11 | 0.228 |
| Uqcrq | 22272 | ubiquinol-cytochrome c reductase, complex III subunit VII | 0.93 | 0.503 | 1.05 | 0.627 | 1.07 | 0.547 |
| Atp6v1e1 | 11973 | ATPase, H+ transporting, lysosomal V1 subunit E1 | 1.19 | 0.0214 | 1.09 | 0.228 | 1.06 | 0.434 |
| Cox10 | 70383 | cytochrome c oxidase assembly protein 10 | 0.88 | 0.241 | 0.92 | 0.405 | 1.05 | 0.632 |
| Atp6v0a2 | 21871 | ATPase, H+ transporting, lysosomal V0 subunit A2 | 1.00 | 0.955 | 0.93 | 0.175 | 1.03 | 0.617 |
| Ndufb3 | 66495 | NADH dehydrogenase (ubiquinone) 1 beta subcomplex 3 | 0.88 | 0.334 | 0.83 | 0.122 | 1.03 | 0.815 |
| Sdhd | 66925 | succinate dehydrogenase complex, subunit D, integral membrane protein | 1.00 | 0.969 | 1.08 | 0.179 | 1.01 | 0.838 |
| Atp5o | 28080 | ATP synthase, H+ transporting, mitochondrial F1 complex, O subunit | 0.75 | 0.00647 | 1.12 | 0.211 | 0.98 | 0.861 |
| Atp6v0a1 | 11975 | ATPase, H+ transporting, lysosomal V0 subunit A1 | 0.91 | 0.0536 | 1.02 | 0.693 | 0.95 | 0.271 |
| Ndufs2 | 226646 | NADH dehydrogenase (ubiquinone) Fe-S protein 2 | 0.95 | 0.346 | 0.94 | 0.281 | 0.91 | 0.0854 |
| Ndufb2 | 68198 | NADH dehydrogenase (ubiquinone) 1 beta subcomplex, 2 | 0.88 | 0.207 | 1.11 | 0.289 | 0.91 | 0.357 |
| Atp6v0d1 | 11972 | ATPase, H+ transporting, lysosomal V0 subunit D1 | 0.97 | 0.639 | 1.01 | 0.846 | 0.88 | 0.0294 |
| Atp5g3 | 228033 | ATP synthase, H+ transporting, mitochondrial F0 complex, subunit C3 (subunit 9) | 0.78 | 0.00857 | 0.99 | 0.879 | 0.85 | 0.0682 |
| Ndufs1 | 227197 | NADH dehydrogenase (ubiquinone) Fe-S protein 1 | 1.04 | 0.459 | 0.96 | 0.468 | 0.85 | 0.00356 |
| Uqcrb | 67530 | ubiquinol-cytochrome c reductase binding protein | 0.93 | 0.558 | 0.96 | 0.775 | 0.83 | 0.162 |
| Ppa1 | 67895 | pyrophosphatase (inorganic) 1 | 0.88 | 0.18 | 1.06 | 0.493 | 0.81 | 0.0225 |
| Ppa2 | 74776 | pyrophosphatase (inorganic) 2 | 0.98 | 0.803 | 0.99 | 0.905 | 0.81 | 0.0195 |
| Cyc1 | 66445 | cytochrome c-1 | 0.80 | 0.0234 | 0.92 | 0.364 | 0.81 | 0.026 |
| Ndufv2 | 72900 | NADH dehydrogenase (ubiquinone) flavoprotein 2 | 1.01 | 0.914 | 1.04 | 0.584 | 0.80 | 0.00153 |
| Atp5g1 | 11951 | ATP synthase, H+ transporting, mitochondrial F0 complex, subunit C1 (subunit 9) | 0.86 | 0.253 | 0.99 | 0.923 | 0.76 | 0.0444 |
| Atp5e | 67126 | ATP synthase, H+ transporting, mitochondrial F1 complex, epsilon subunit | 0.98 | 0.917 | 1.28 | 0.116 | 0.75 | 0.089 |
| Atp6v1d | 73834 | ATPase, H+ transporting, lysosomal V1 subunit D | 0.99 | 0.866 | 0.99 | 0.839 | 0.75 | 6.23E-06 |
| Ndufa1 | 54405 | NADH dehydrogenase (ubiquinone) 1 alpha subcomplex, 1 | 1.11 | 0.319 | 1.19 | 0.0875 | 0.75 | 0.012 |
| Cox7a1 | 12865 | cytochrome c oxidase subunit VIIa 1 | 0.35 | 0.00384 | 0.30 | 0.00064 9 | 0.74 | 0.306 |
| Atp6v0c | 11984 | ATPase, H+ transporting, lysosomal V0 subunit C | 0.85 | 0.436 | 0.64 | 0.0413 | 0.73 | 0.166 |
| Uqcrfs1 | 66694 | ubiquinol-cytochrome c reductase, Rieske iron-sulfur polypeptide 1 | 0.90 | 0.133 | 0.92 | 0.205 | 0.71 | 1.31E-05 |
| Cox6a1 | 12861 | cytochrome c oxidase subunit VIa polypeptide 1 | 0.90 | 0.32 | 1.03 | 0.79 | 0.69 | 0.00148 |
| Uqcr10 | 66152 | ubiquinol-cytochrome c reductase, complex III subunit X | 0.98 | 0.883 | 1.19 | 0.0881 | 0.69 | 0.00146 |
| Cox5a | 12858 | cytochrome c oxidase subunit Va | 0.90 | 0.208 | 0.91 | 0.203 | 0.63 | 4.40E-07 |
| Cox7b | 66142 | cytochrome c oxidase subunit VIIb | 0.89 | 0.189 | 1.00 | 0.994 | 0.59 | 4.59E-07 |
| Cox17 | 12856 | cytochrome c oxidase assembly protein 17 | 0.89 | 0.242 | 0.94 | 0.486 | 0.57 | 3.67E-06 |
| Ndufa5 | 68202 | NADH dehydrogenase (ubiquinone) 1 alpha subcomplex, 5 | 0.86 | 0.157 | 0.97 | 0.733 | 0.56 | 6.26E-06 |
| Cox7c | 12867 | cytochrome c oxidase subunit VIIc | 0.72 | 0.0364 | 0.96 | 0.771 | 0.47 | 1.15E-05 |

**Sakaguchi et al., Supplementary Table 6**

## Primer Sequences for Quantitative Real-Time PCR

| Gene | Direction | Sequence |
|---|---|---|
| TBP | Forward | ACCCTTCACCAATGACTCCTATG |
| TBP | Reverse | TGACTGCAGCAAATCGCTTGG |
| FoxK1 | Forward | ACCCACGAATAGCTTGACGG |
| FoxK1 | Reverse | GCATTAGCGGCTACTGAGACG |
| Vcam1 | Forward | CGGGAA AGCCTCGACTTCCA |
| Vcam1 | Reverse | TTATGTCTATGAGCTTTGTA |
| AFF4 | Forward | GGGATCGCCCGACGCGTCCT |
| AFF4 | Reverse | ACTTGAATGTCTCTAACCGG |
| Tgoln1 | Forward | TCGAAGGCTTTTTGCTCCGG |
| Tgoln1 | Reverse | GTCTATAGTTTCGTAACTTT |
| ALPL | Forward | CCCGCCCCTCCGGCCGCCCA |
| ALPL | Reverse | GGTTTATAAAGACTCCGGTC |
| Mcam | Forward | TGCGCCCCGCCCCGGCCCGC |
| Mcam | Reverse | CACAATAACAAGGTTTTCGT |
| Adgrg1 | Forward | AGTTCTCCTCGAGTCGGTAC |
| Adgrg1 | Reverse | CGTCGTGTCAAACCTCTCAA |
| SGK1 | Forward | CCTTGGATGTGTTAGTTTTT |
| SGK1 | Reverse | AACTTTTGATAAACGTATTT |
| GAPDH | Forward | GTATTGGGCGCCTGGTCACC |
| GAPDH | Reverse | CGCTCCTGGAAGATGGTGATG |
| mt-Nd1 | Forward | TGCCAGCCTGACCCATAGCC |
| mt-Nd1 | Reverse | ATGGGCCGGCTGGGTATTCT |
| mt-Nd6 | Forward | CCCGCAAACAAAGATCACCCAGC |
| mt-Nd6 | Reverse | GTTGGAAGGAGGGATTGGGGTAGC |

**Sakaguchi et al., Supplementary Table 7**

# SiRNA Sequences

| Dharmacon On Target plus SMART pool SiRNA | | | |
|---|---|---|---|
| Gene | Species | Name | Target Sequence |
| FoxK1 | mouse | siFoxk1#1 | 5'-GCAUGGGCCUGUCGAGCUU |
| FoxK1 | mouse | siFoxk1#2 | 5'-UCGAGUUCCUGAUGCGACA |
| FoxK1 | mouse | siFoxk1#3 | 5'-GAACAAGCAUUCCGAAAGC |
| FoxK1 | mouse | siFoxk1#4 | 5'-UCACAUCGCUAUACCAUAA |
| FoxK2 | mouse | siFoxk2#1 | 5'-UGUCAUCAUCAUAACGUUA |
| FoxK2 | mouse | siFoxk2#2 | 5'-CGGAAUGUCUGUCUCGUAA |
| FoxK2 | mouse | siFoxk2#3 | 5'-UCACAGAGUAGCCGUAGUA |
| FoxK2 | mouse | siFoxk2#4 | 5'-CCAGAGCUCAAGCGAGUUA |
| GSK3β | mouse | siGSK3β#1 | 5'-GGACCCAAAUGUCAAACUA |
| GSK3β | mouse | siGSK3β#2 | 5'-CCACAGGAAGUCAGUUAUA |
| GSK3β | mouse | siGSK3β#3 | 5'-UCAGAAGUCUAGCCUAUAU |
| GSK3β | mouse | siGSK3β#4 | 5'-GAUUACACGUCCAGUAUAG |
| Dharmacon On Target plus Non-targeting pool SiRNA | | | |
| Non-targeting | | siControl#1 | 5'-UGGUUUACAUGUCGACUAA |
| Non-targeting | | siControl#2 | 5'-UGGUUUACAUGUUGUGUGA |
| Non-targeting | | siControl#3 | 5'-UGGUUUACAUGUUUUCUGA |
| Non-targeting | | siControl#4 | 5'-UGGUUUACAUGUUUUCCUA |

**Sakaguchi et al., Supplementary Table 8**
